# Local accumulation of very long-chain PUFA in plexiform layers associates with retinal dysfunction in a mouse model of peroxisomal ACBD5-deficiency

**DOI:** 10.1007/s00018-025-05971-8

**Published:** 2025-12-01

**Authors:** Julia Merz, Elisabeth Müller, Warda Darwisch, Richard Fairless, Yixin Wang, Silke Vorwald, Sharau Darwisch, E. Ronald Curticean, Feng Shao, Irene Wacker, Rasmus R. Schröder, Claudia Pitzer, Christian Schultz, Jan-Bert van Klinken, Frederic M. Vaz, Frank Kratzer, Kathrin Schwarz, Jürgen G. Okun, Yuxi Feng, Carsten Hopf, Markus Islinger

**Affiliations:** 1 Institute of Neuroanatomy, Medical Faculty Mannheim, Heidelberg University, Heidelberg, Germany; 2 Mannheim Center for Translational Neuroscience (MCTN), Medical Faculty Mannheim, Heidelberg University, Heidelberg, Germany; 3 Center for Mass Spectrometry and Optical Spectroscopy (CeMOS), Technische Hochschule Mannheim, Mannheim, Germany; 4 Medical Faculty Heidelberg, Heidelberg University, Heidelberg, Germany; 5 Department of Neurology, University Clinic Heidelberg, Heidelberg, Germany; 6 Clinical Cooperation Unit (CCU) Neurooncology German Cancer Consortium (DKTK), German Cancer Research Center (DFKZ), Heidelberg, Germany; 7 Experimental Pharmacology, European Center for Angioscience (ECAS), Medical Faculty Mannheim, Heidelberg University, Heidelberg, Germany; 8 BioQuant, Medical Faculty Heidelberg, Heidelberg University, Heidelberg, Germany; 9 Interdisciplinary Neurobehavioral Core, INBC, Ruprecht-Karls-Universität Heidelberg, Heidelberg, Germany; 10 Department of Laboratory Medicine and Pediatrics, Laboratory Genetic Metabolic Diseases, Amsterdam UMC location University of Amsterdam, Emma Children’s Hospital, Amsterdam, The Netherlands; 11 Core Facility Metabolomics, Amsterdam UMC Location University of Amsterdam, Amsterdam, The Netherlands; 12 Department of Human Genetics, Leiden University Medical Center, Leiden, Netherlands; 13 Amsterdam Gastroenterology Endocrinology Metabolism, Inborn errors of metabolism, Amsterdam, The Netherlands; 14 Department of General Pediatrics, Division of Neuropediatrics and Metabolic Medicine, University Hospital Heidelberg Center of Pediatric and Adolescent Medicine, Heidelberg, Germany; 15 Institute of Neuroanatomy, Mannheim Center for Translational Neuroscience (MCTN), Medical Faculty Mannheim, Heidelberg University, Ludolf-Krehl Str. 13-17, Mannheim, 68167 Germany

**Keywords:** Peroxisomes, Metabolic disorders, Retinodystrophy, Very long-chain fatty acids (VLCFA), RDLKD (ACBD5-deficiency)

## Abstract

Patients deficient in the peroxisomal membrane protein ACBD5 regularly exhibit a dystrophy of the retina along with decline in visual acuity. Despite the prevalent retinal phenotype, information on the pathogenesis of the retinodystrophy is limited. To gain insight into the cellular, subcellular and molecular alterations occurring in the retina, we analyzed an ACBD5-deficient mouse model by immunofluorescence microscopy, electron microscopy, full-field electroretinography (ffERG) and as well as analytical and spatial mass spectrometry (MS)-based lipidomics techniques. Histological results implied that ACBD5-deficient mice exhibit a moderate degeneration of photoreceptor, bipolar, ganglion and retinal pigment epithelial cells accompanied, however, by a prominent activation of astroglia and microglia. Reduced a- and b-wave amplitudes from ffERG point to a severe functional dysregulation of retinal signal transduction with a focus at the level of the information-processing cell of the inner retina. At the lipidome level, very long-chain polyunsaturated fatty acids (VLC-PUFA) accumulated in phosphatidylcholines from retina homogenates, most likely disrupted by a decline in peroxisome functions. Remarkably, as revealed by MALDI MS imaging, these lipidome changes affected neither the whole retina nor the photoreceptor outer segments (POS), where VLC-PUFAs display the highest concentration in phospholipids of POS membrane discs. In contrast, VLC-PUFAs in ACBD5-deficient mice consistently accumulated in the inner retinal region from the outer (OPL) to inner plexiform layer (IPL). In line with VLC-PUFA-accumulations, photoreceptor ribbon synapses in the OPL showed morphological signs of degeneration on the ultrastructural level. Hence, peroxisomal dysfunction appears to affect cell type-specific lipid homeostasis, thereby disrupting local retinal membrane physiology leading to a severe neuroinflammation of the ACBD5-deficient mouse retina.

**Supplementary Information:**

The online version contains supplementary material available at 10.1007/s00018-025-05971-8.

## Introduction

Peroxisomes are ubiquitous, small, membrane-bound and mostly spherical organelles [1]. Functionally, they contribute to several pathways of lipid metabolism such as the degradation of very long-chain and branched-chain fatty acids as well as the synthesis of ether lipids and distinct ω6- and ω3-polyunsaturated fatty acids such as docosahexaenoic acid (DHA) [2]. Moreover, they are involved in the defense against reactive oxygen species (ROS), viruses and the mediation of inflammatory responses. Inherited disorders of peroxisomal metabolism and biogenesis are generally characterized by severe degenerative alterations in the central nervous system [3]. Since the optic part of the eye differentiates from a vesicular outgrowth of the brain and exhibits a highly specialized membrane lipidome, even milder forms of peroxisomal disorders often present with a retinal pathology [4]. However, unlike for patients from the Zellweger spectrum [5, 6], data describing the ophtalmological pathology in peroxisomal single-enzyme deficiencies is still scarce and often restricted to information from noninvasive techniques such as fundoscopy and electroretinograms (ERG). Mouse models can be valuable tools to delineate the pathogenesis of peroxisome disorders. However, descriptions of the retinal degeneration in mutant mice are largely confined to studies from *Mfp2*^*−/−*^ (Multifunctional Protein 2), *Acox1*^*−/−*^ knockout mice, Pex1(G843D) knockin mice and to a lesser extent *Gnpat*^*−/−*^ (glycerone-phosphate O-acyltransferase) mice [7–10]. Patients lacking the peroxisomal tail-anchored membrane protein ACBD5 (acyl-CoA binding domain-containing protein 5) have been first detected in a genome screen for uncharacterized retinopathies [11]. While patients with an ACBD5-deficiency show severe brain pathologies, all hitherto described patients also exhibit a prominent retinopathy [12–17], leading to an annotation “Retinal Dystrophy with Leukodystrophy” (RDLKD) in the OMIM database. Functionally, ACBD5 not only assists in the import of very long chain acyl-CoAs into peroxisomes for subsequent β-oxidation [18, 19], but also facilitates membrane contacts to the endoplasmic reticulum (ER) by interaction with the ER membrane resident VAPA/B (vesicle-associated membrane protein-associated protein A/B) and to mitochondria by interacting with PTPIP51 [20–22]. These interactions are likely relevant for the exchange of pathway intermediates, membrane lipids reactive oxygen species and signals between the organelles [12], which may contribute to the development of a complex RDLKD pathology. Two ACBD5-deficient mouse models have been described recently [23, 24]. Both exhibit a prominent motor deficiency, accompanied by a cerebellar degenerative and inflammatory pathology. Moreover, retinal pathologies were reported for both mouse models but were not yet extensively characterized [23, 24]. Hence, in order to unravel the pathogenesis behind the retinodystrophy observed in human RDLKD patients, we investigated an C57BL/6J-A^tm1Brd^ Acbd5^tm1a(EUCOMM)Wtsi/WtsiCnbc^ mouse line (in the following termed *Acbd5*^*−/−*^), in which Acbd5 transcription is blocked by insertion of a neo cassette between exon 2 and exon 3 [25], to gain mechanistic information on its retinal pathology. Notably, the retina exhibits a highly specialized membrane lipid composition, in which DHA, very long-chain fatty acids (VLCFA) and ether lipids are found in unusually high concentrations [26–28]. Elevated levels of VLCFA are a hallmark in RDLKD [18, 19] and fatty acids (FA) with a chain length above C30 are highly enriched in phosphatidylglycerols of the CNS of the *Acbd5*^*−/−*^ strain [23]. The fatty acid elongase ELOVL4 elongates polyunsaturated FA above a chain length of C32. Interestingly, mutations in ELOVL4 lead to macular degeneration implying that a highly balanced homeostasis of the VLC-PUFA composition in retinal membranes is crucial for a healthy retina [29, 30].

In this work, we combined a detailed morphological analysis of the retina with multifocal electroretinography (mfERG) and an extensive and spatial lipidomics analysis to gain information on potential lipid alterations in this highly stratified tissue. Morphologic analysis revealed degenerative cell alterations at all levels of the murine retina including retinal pigment epithelium (RPE), photoreceptor cells, bipolar cells and ganglion cells, which were accompanied by a highly significant increase of polyunsaturated VLCFA (VLC-PUFA) in phosphatidylcholines (PC). Remarkably and in line with the results from ERGs and inflammatory parameters, MALDI MS imaging revealed that the VLC-PUFA accumulation was restricted to the inner retinal layers overlapping best with the localization of bipolar cells. Hence, bipolar cell metabolism appears to be most susceptible to the peroxisomal changes induced by the lack in ACBD5.

## Materials and methods

### Animal breeding

The C57BL/6N-A^tm1Brd^ Acbd5^tm1a(EUCOMM) Wtsi/WtsiCnbc^ mouse strain was produced at the Sanger Institute. In this mouse, *Acbd5* gene transcription is blocked by inserting a neo cassette between exon 2 and exon 3 using the so-called knock-in first technology [25]. Sperm from *Acbd5*^+/−^ mice was received from the European Mouse Mutant Archive (EMMA) and used for fertilization of wild-type C57BL/6J mice (*Acbd5*^*+/+*^). Heterozygous mice were identified by PCR (Table S1) and consecutively intercrossed into the C57BL/6J background to yield homozygous the *Acbd5*^*−/−*^ and *Acbd5*^*+/+*^ mice. As the *Acbd5*^−/−^ mice are fertile, homozygous *Acbd5*^*−/−*^ and correspondent *Acbd5*^*+/+*^ mice were further bred as individual strains (breeding & experiment permission under 35–9185.81.81/G-202/19, Regierungspräsidium Karlsruhe, Germany). Occasionally, both strains were interbred and outcrossed to avoid genomic drift. The animals were kept according to the guidelines for care and use of laboratory animals of Germany at the animal facilities of the Medical Faculty Mannheim, Heidelberg University at a 12 h/12 h light cycle and were fed ad libitum with standard rodent chow.

### Tissue preparation

In order to receive retina preparations for the microscopic analysis, mice aged 3 and 12 months were either sacrificed by cervical dislocation for immersion fixation or anesthetized with xylazin/ketamine at a concentration of 16/120 mg/kg body weight for perfusion fixation. For the latter, the thorax was opened immediately after narcosis and perfusion was performed via the left heart ventricle first removing blood with 0.9% NaCl for 5 min and subsequently fixing the tissue for 10 min with 4% paraformaldehyde (PFA) in phosphate buffered saline (PBS, pH 7.4). For both fixation methods, eyes were carefully removed from the orbita and for better handling immersed in 4% PFA in PBS until further preparation. For each light microscopy analysis, 4–6 animals per genotype were analyzed, for electron microscopy (EM), 3–4 animals/genotype were investigated.

### Light microscopy

In order to prepare the tissue for sectioning, eyes were opened by a small incision in the cornea. For cryofixation lens and vitreous body were carefully removed. Subsequently, all eye bulbs were immersed in 4% PFA for 15 min (cryoconservation) or overnight (paraffin). Thereafter, the fixed eyes were either embedded in Tissue-Tek O.C.T. compound (Sakura Finetek, Umkirch, DE), submersed in isopentane and frozen in liquid nitrogen or in paraffin after dehydration by incubation in an ethanol/PBS series (70, 80, 90, 96, 99%). Cryo-embedded tissue was cut into 10 μm sections using a Leica CM3050 cryostat (Wetzlar, DE), mounted onto microscope slides and stored at −20 °C until performing the immunostaining. Paraffin embedded tissue was cut in 5–10 μm sections using a conventional microtome and subjected to hematoxylin/eosin (HE) staining. Transverse sections at the eye equator indicated by the entry of the optic nerve were used for measurement of retinal layer thickness. Ten measurements were performed in a 75° angle towards each side of the optic nerve entry and used for calculation of the average retinal layer thicknesses. The ONL/INL ratio was calculated by dividing diameters [µm] of the respective retinal layers.

For RPE flat mount preparations, eye bulbs were cut at the ora serrata and the cornea, lens and vitreous body were excised. Subsequently, the retina was carefully removed from the RPE and the remaining eye bulb was cut crosswise to yield a “shamrock” of four retinal quadrants. Subsequently, the RPE flat-mounts were fixed with 4% PFA in PBS for 20 min. After fixation, all flat mount preparations were subjected directly to immuno- and/or lipid staining. For determination of the RPE cell size and nucleus number, 10 images of 45,000 µm^2^ from central and peripheral regions of the four leaflets were used per specimen. For quantification of lipid droplets (LD), rhodopsin and cone opsin-positive vesicle numbers, 5 images per 45,000 μm from close to the center and in the middle of each quadrant were used for each individual.

### Immunofluorescence staining

Cryo-conserved sections were thawed to room temperature and post-fixed with 4% PFA for another 10 min. After three 5 min washing steps in PBS the tissue was incubated in a combined blocking/permeabilization step for 30 min in 1% bovine serum albumin, 0.2% fish skin gelatin, 0.1% Triton X-100. Overnight the specimens were incubated with primary antibodies diluted in blocking solution (Table S1) at 4 °C. After washing for three times in PBS, the tissue sections were incubated with secondary antibodies (Table S2) and in case of nuclear staining TO-PRO-3 in PBS (Thermo Fisher, 1:1000) for 1.5 h at room temperature. After three final 5 min washing steps in PBS, the slides were rinsed with A. dest. and covered with Roti^®^-Mount (Carl-Roth GmbH, Karlsruhe, DE) and a cover slip. RPE whole mounts were stained in immersion according to the same protocol. For lipid staining 0.5 g of Nile Red was dissolved in 1 mL acetone. This stock was added in a 1:100 dilution to the secondary antibody/PBS solution.

Microscopy analysis was performed with a C2 Nikon confocal microscope equipped with 488 nm, 561 nm and 647 nm laser lines and either an ApoPlan 20 × (0.75 NA), ApoPlan 60x (oil immersion, 1.4 NA) or a ApoPlan 100× (oil immersion, 1.45 NA) objective. Images were acquired in stacks of 5–15 planes with a distance of 1 μm. For the detection of Nile Red, 488 and 561 lasers were used for excitation of neutral lipid and phospholipid fluorescence with emission detection windows set to band widths of 560–606 nm and 630–670 nm, respectively. For the local quantification of microglia, IBA1-positive cells were counted across whole retina transects in 5 individuals per genotype. Ribbon synapses from the OPL and IPL were quantified by anti-ribeye staining in 5 planes from two 45,000 µm^2^ image stacks. Images were taken at an angle of approx. 30° from the *N. opticus* entry. For quantification of GFAP percentage areas three 45.000 µm^2^ confocal stacks per specimen (*n* = 4–6/genotype) were used. For quantification of the remaining retinal cell type numbers and subcellular structures, 5 images per 45,000 µm^2^ covering representative regions from the whole retina radius excluding the areas next to the *ora serrata* and optic nerve entry were used per each specimen (*n* = 4–6 per genotype and age). For quantitative image analysis the open-source software ImageJ2 (Version 2.13.1) was applied. Nested Student’s T or Anova Tests were performed for statistical analysis using Graph Pad Prism. Optimization of image contrast and formats for figures was performed with Adobe Photoshop CS2 without changing the original image parameters.

### Electron microscopy

For electron microscopy (EM), eye bulbs were opened by four crosswise incisions in order to obtain flat mount retina preparations and fixed overnight in 1.25% glutaraldehyde in 150 mM cacodylate, pH 7.2 at 4 °C. For the peroxisome labeling, the specimens were washed in cacodylate buffer (150 mM cacodylate, 0.05% CaCl_2_, pH7.2), incubated for 1 h in 0.2% diaminobenzidine (DAB) in Teorell Stenhagen buffer (TS, 50 mM phosphoric acid, 57 mM boric acid 35 mM citric acid, 345 mM NaOH, pH 10.5) and for another hour in 0.2% DAB in TS containing 0.15% H_2_O_2_. Subsequently, the tissues were again incubated overnight in 1.25% glutaraldehyde in cacodylate buffer. After a washing step in cacodylate buffer, the specimens were incubated in 0.5% OSO4/0.8% K_4_[Fe(CN)_6_]·3H_2_O in a microwave with 5 pulses for 10 s and for another 30 min at room temperature. After two washing steps in A. dest. the samples were contrasted overnight with 2% uranyl acetate in 25% ethanol, 4 °C. For embedding, the samples were dehydrated in a series of increasing ethanol concentrations (50, 70, 96, 99.9%) followed by incubation in a 1:2 mixture of water-free acetone and epon 812 (42.4 g glycidether 100, 29.6 g 2-dodecenyl succinyl anhydride, 18.4 g methyl nadic anhydride) in 10 min intervals. The retinae were incubated overnight at 4 °C in epon 812 and polymerized at 63 °C adding approximately 50 µl of a benzyl dimethylamine/tri-dimethylaminomethyl phenol mixture per mL resin.

After complete polymerization ultrathin sections of 70–100 nm were cut with an Ultracut-S Ultramicrotome (LEICA, Wetzlar, DE) and mounted onto silicon-wafers (Si-MAT Silicon Materials, Kaufering, DE). Contrast in the sections was enhanced by incubation in 5% uranyl acetate for 5–10 min and Reynolds lead citrate solution for 0.5–2 min. The sections were imaged in a field emission scanning electron microscope (Ultra 55, Carl Zeiss Microscopy, Oberkochen, DE) at 1.5 keV primary electron energy using the SE2 detector for secondary electrons simultaneously with the ESB detector for backscattered electrons and the Atlas 5 software for automated acquisition of large scan fields of the regions of interest. Images were acquired at resolutions between 3 and 6 nm/pixel).

### Lipidomics analysis

For lipidome analysis, eye bulbs from eight 12-month-old *Acbd5*^*−/−*^ and eight age-matched *Acbd5*^*+/+*^ mice were opened at the ora serrata, the neural retinae removed and immediately snap-frozen in liquid nitrogen. Until lipid extraction, the tissue was stored at −80 °C. Retina homogenates were prepared in Milli-Q water using a Qiagen Tissuelyser II with 5 mm stainless steel beads (2 × 30 s, 30 Hz). Protein concentration was measured via BCA assay. Lipidomics analysis, including lipid extraction, separation, and quantification, was performed as published recently [31]. In brief, lipids were extracted using 1:1 chloroform: methanol with internal standards for lipid major classes. Analysis was conducted on a Thermo Scientific Ultimate 3000 HPLC coupled to a Q Exactive Plus Orbitrap mass spectrometer, employing normal and reversed-phase chromatography in both negative and positive ion mode. Data processing and lipid annotation were performed using an in-house lipidomics pipeline in R and MATLAB, as described [31].

### Full field ERG and optical coherence tomography (OCT) imaging

To evaluate neuronal function in the retina, ffERG and OCT was performed on mice *n* = 8–9 per genotype and per experimental approach.

Full-field ERGs were performed on *Acbd5*^*−/−*^ and *Acbd5*^*+/+*^ mice according to previously published procedures [32, 33]. Mice were dark adapted overnight and anesthetized with ketamine (150 mg/kg) and xylazine (10 mg/kg), and the pupils were dilated with 0.5% atropine (Ursapharm, Saarbrücken, Germany). ERGs were recorded using the UTAS Visual Diagnostic System (LKC Technologies, Gaithersburg, MD, USA) and a contact lens electrode with gold contact (LKC Technologies). The electrical contact, as well as prevention of eye desiccation, was facilitated through application of Liquifilm OK (Allergan, Westport, Ireland). Reference and ground needle electrodes were placed subcutaneously in the neck and tail, respectively. A heating pad controlled by a rectal temperature probe was maintained at 37 °C. A single eye per mouse was recorded from and taken as an individual data point. Functional analyses included single-flash ERGs under scotopic (dark-adapted, no background illumination, 0 cd·s/m^2^) and photopic (light adaptation with background illumination of 30 cd/m^2^, beginning 10 min before recording) conditions. Single white flash intensity series ranged from − 4.0 to + 1.5 log cd·s/m^2^ for scotopic recordings, and − 1.5 to + 1.5 log cd·s/m^2^ for scotopic recordings. A total of 10 responses were averaged with interstimulus intervals of 5 (for − 4.0 to − 0.5 log cd·s/m^2^) or 17 (for 0 to 1.5 log cd·s/m^2^) seconds. For all ERG recordings, band-pass cutoff frequencies were 0.3 and 300 Hz. To determine full-field flash ERG responses with increasing frequency stimulation, dark-adapted mice were presented with 0 log cd·s/m^2^ intensity flashes using a flicker ERG protocol at frequencies ranging from 1 to 20 Hz, again using the UTAS Visual Diagnostic System. One-second recordings were made and averaged over 30–50 recordings (sampling rate, 1000 Hz).

For optic coherence tomography (OCT), mice were anesthetized via intraperitoneal injection of a mixture of ketamine (75 mg/kg) and xylazine (3.75 mg/kg), with the mice kept on a heating pad at 37 °C during the procedure. Pupillary dilation was achieved by applying a topical drop of 0.5% tropicamide before test. The mice were positioned in front of a scanning laser ophthalmoscope (RETImap, Roland Consult, Brandenburg an der Havel, Germany), with a DTL electrode placed on the cornea. A + 100-diopter contact lens (Roland Consult, Brandenburg, Germany), coupled with 2% Methocel gel, ensured proper corneal contact and hydration. OCT B-scan images were acquired to examine the retinal structure. The total retinal thickness (TRT) was defined as the width from the retinal nerve fiber layer (RNFL) to the RPE. Eight or five measurements were taken at the same distance (600–800 μm) from ONH and averaged to calculate the average TRT as well as IPL, INL and OPL diameters.

### Measurement of DHA blood plasma concentration

For DHA plasma determination, 3-month-old (*n* = 4/genotype) and 12-month-old (*n* = 6/genotype) *Acbd5*^−/−^ and *Acbd5*^+/+^ mice were sacrificed by cervical dislocation and blood taken from the portal vein. The blood was incubated for 30 min at RT and subsequently pelleted at 850 × g for 5 min to obtain the plasma. The plasma DHA was converted to DHA-methyl ester using methanolic HCl (3 N). To this end 70 µl of plasma was mixed with methanolic HCl under vortexing and incubated for 1 h at 80 °C., The obtained DHA-methyl ester was extracted with n-Hexan and analysed by gas chromatography-flame ion detection (GC-FID, 7890 A, Agilent Technologies, USA) using nonadeconoic acid-methyl ester (C19:0) as internal standard.

### MALDI-MS imaging of lipids

For matrix-assisted desorption/ionization mass spectrometry imaging (MALDI-MSI) of lipids [34], 12 month-old animals were sacrificed by cervical dislocation, eye bulbs excised and embedded in 7.5% hydroxypropyl methylcellulose, 2.5% polivinylpyrroliidone by immersion in liquid nitrogen. For localization of the mass-to-charge (m/z)-values to specific regions of the retina, transverse serial 10 μm sections from the eyes close to the equator were cut with a cryostat (−20 °C) and sequentially mounted on conductive indium tin oxide (ITO) coated glass slides (Bruker Daltonics, Bremen, Germany) for MS imaging and conventional microscope slides for HE staining. HE stains were performed as described above.

For matrix spray-coating a 2,5-dihydroxyacetophenon (DHAP, Thermo, USA) matrix solution (10 mg/mL) was prepared in ACN/H_2_O/TFA (70:30:0.1, v/v), sonicated for 15 min and deposited in ten spraying cycles onto slides using an M5 Sprayer (HTX Technologies LLC, Chapel Hill, USA). The spraying parameters were as follows: nozzle temperature 75 °C; and bed temperature at 30 °C; flow rate at 0.1 mL/min with a nozzle velocity of 1200 mm/min. The track spacing was maintained at 3 mm in a HH pattern. The height of the nozzle from the slide surface was set at 40 mm. Following matrix-coating, slides were immediately placed in on a tims-TOF fleX MS (Bruker Daltonics) equipped with a smartbeam 3D 10 kHz laser. Data was acquired in positive ionization mode (m/z range of 300–2000) with 200 laser shots per pixel, 10 kHz laser frequency and lateral step size of 10 μm. The Ion Transfer parameters were as follows: MALDI Plate Offset 50 V, Deflection 1 Delta 70 V, Funnel 1 RF 420 Vpp, isCID Energy 0.0 V, Funnel 2 RF 350 Vpp, and Multipole RF 320 Vpp. Collision Cell parameters: Collision Energy 10 eV, and Collision RF 2000 Vpp. Quadrupole parameters: Ion Energy 5 eV, and Low Mass m/z 320. Focus Pre TOF parameters: Transfer Time 85 µs, and Pre Pulse Storage 10 µs. For external calibration of the trap unit of the timsTOF fleX mass spectrometer, ESI-L Low Concentration Tuning Mix (Agilent Technologies, Waldbronn, Germany) was used.

The MALDI-TOF MSI raw data was imported into SCiLS Lab (v. 2024, Bruker Daltonic). The imaging data were total ion current (TIC) normalized. The MSI data were converted to the open source.imzML-file and uploaded to the false discovery rate (FDR) controlled Metaspace database for annotation using SwissLipids (v 2018-02−02) and Lipid Maps (v 2017-12−12). Lipid species PC 38:6, PC 40:7, PC 42:9 and PC 54:12 were annotated with a FDR of ≤ 11%. Ion images of lipid species accumulating specifically in the *Acbd5*^−/−^ mice were selected based on analysis of the prior acquired LC MS/MS data. Lipid species with a fold change higher than 20 between *Acbd5*^+/+^ and *Acbd5*^−/−^were selected as potential lipid candidates. Respective ion images of PC(54:12), PC(58:9), PC(60:9), PC(60:10) and PC(62:10) were manually collected and exported as.tiff-files from SCiLS Lab. Lipid annotation was done by matching m/z-signals with MS1-level database (SwissLipids) within 5 ppm mass tolerance and the obtained LC MS/MS at < 10 ppm mass tolerance. For statistical evaluation, relative abundance of lipid candidates was quantified by comparing mean values from 5 different biological replicates per genotype using unpaired t-test with Benjamini-Hochberg correction.

## Results

RDLKD patients lacking the peroxisomal membrane protein ACBD5 suffer from retinal degeneration described either as a cone-rod or rod-cone dystrophy. At the metabolic level, RDLKD is characterized by an accumulation of VLCFA in membrane lipids [19, 35], which might induce alterations in membrane physiology initiating the pathogenesis of the disorder. Hence, in order to gain information on its retinal pathogenesis, we correlated cellular alterations in the different layers of the retina with regional changes in the membrane lipid spectrum of *Acbd5*^−/−^ mice.

### The retinal pathology in *Acbd5*^*−/−*^ mice is characterized by inner retinal layer thickening and a moderate decline in information processing cell types

**Fig. 1:**
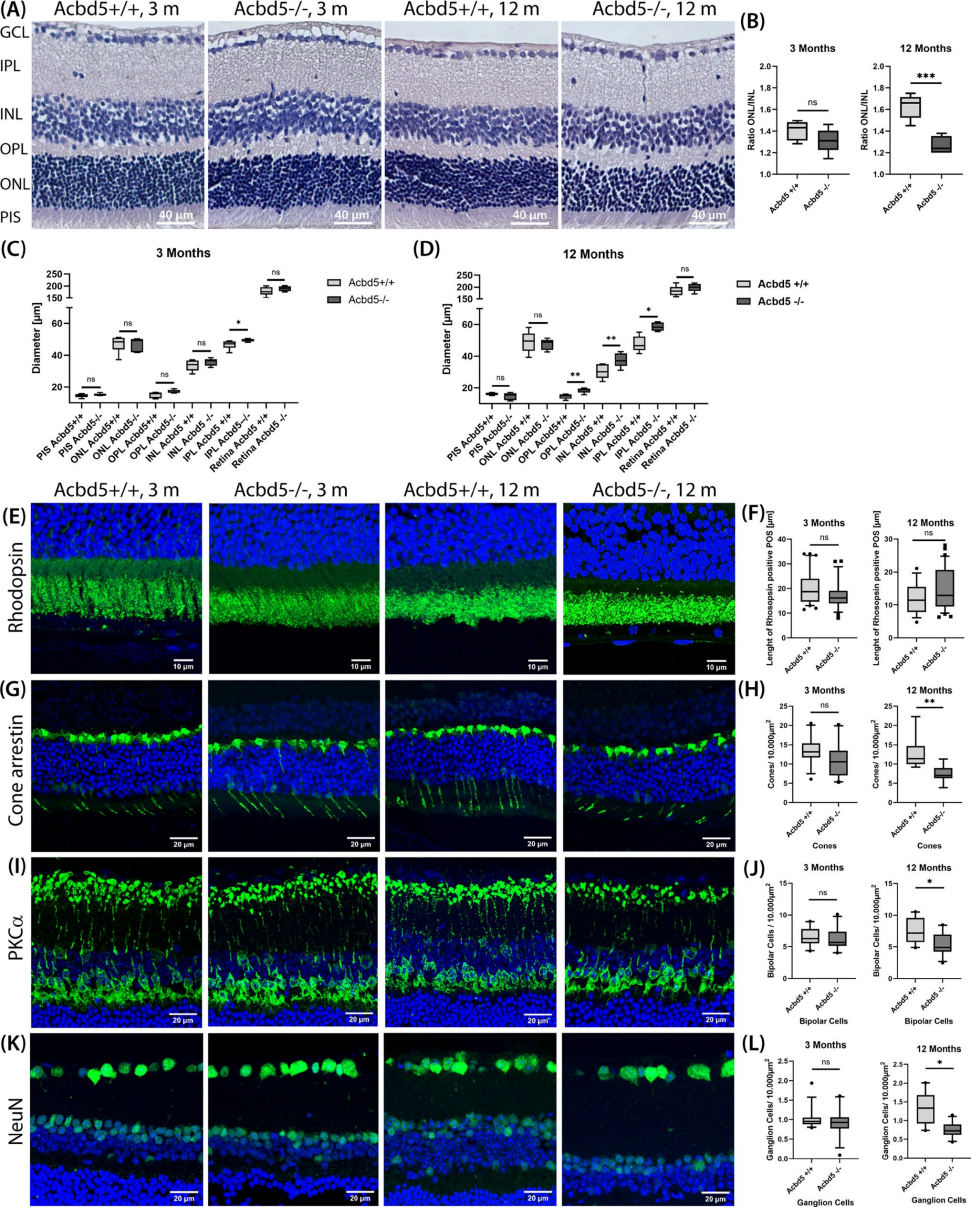
Cellular alterations in the retina of *Acbd5*^*−/−*^ mice. (**A**) Representative images of HE-stained sections from the retina of 3-month (3 m) and 12-month-old (12 m) *Acbd5*^*−/−*^ mice and *Acbd5*^*+/+*^ controls. (**B**) ONL/INL ratio calculated from the HE-stained retinae (3 m: *Acbd5*^*+/+*^*n* = 6, *Acbd5*^*−/−*^*n* = 4; 12 m: *Acbd5*^*+/+*^*n* = 5, *Acbd5*^*−/−*^*n* = 5). (**C**, **D**) Average diameter of individual retinal layers in 3- and 12-month-old *Acbd5*^*−/−*^ and *Acbd5*^*+/+*^ mice according to the HE stained sections. (**E**) IF images from ONL, PIS and POS showing nuclei (blue, TOPRO) and rhodopsin (green). (**F**) Length of POS identified by the rhodopsin signal (3 m: *Acbd5*^*+/+*^*n* = 6, *Acbd5*^*−/−*^*n* = 5; 12 m: *Acbd5*^*+/+*^*n* = 4, *Acbd5*^*−/−*^*n* = 7). (**G**) IF images showing regions from INL to POS; nuclei are visualized by TOPRO (blue), cone photoreceptor cells by antibodies against cone arrestin (green). (**H**) Retinal cell densities for cone photoreceptors identified by cone arrestin (ARR4) (3 m: *Acbd5*^*+/+*^ *n* = 6, *Acbd5*^*−/−*^ *n* = 5; 12 m: *Acbd5*^*+/+*^ *n* = 5, *Acbd5*^*−/−*^*n* = 6). (**I**) Images of retinal ONL to GCL with nuclei stained by TOPRO (blue) and bipolar cells by an antibody against PKCα (protein kinase C alpha) (**J**) Retinal cell densities for bipolar cells identified by PKCα (3 m: *Acbd5*^*+/+*^*n* = 6, *Acbd5*^*−*/−^*n* = 5; 12 m: *Acbd5*^*+/+*^*n* = 4, Acbd5^−/−^*n* = 5). (**K**) IF of retinal ONL to GCL showing nuclei (blue, TOPRO) and ganglion cells (green, NeuN antibody). (**L**) Retinal cell densities for ganglion cells identified by NeuN (3 m: *Acbd5*^*+/+*^*n* = 6, *Acbd5*^*−/−*^*n* = 5; 12 m: *Acbd5*^*+/+*^*n* = 4, *Acbd5*^*−/−*^*n* = 5). Abbr.: ONL – outer nuclear layer, OPL - outer plexiform layer, INL – inner nuclear layer, IPL – inner plexiform layer, POS – photoreceptor outer segments, PIS – photoreceptor inner segments, GCL – ganglion cell layer; ns – not significant, * *p* < 0.05, ** *p* < 0.01, *** *p* < 0.001, **** *p* < 0.0001

Histological investigation of the retina of 12-month-old *Acbd5*^*−/−*^ mice revealed a decreased ratio between outer (ONL) and inner nuclear layer (INL) thickness suggesting a degeneration of photoreceptor cells [23]. To monitor a potential, progressive retinal degeneration we here analyzed retinae from cohorts of 3-month-old and 12-month-old *Acbd5*^*−/−*^ mice (Fig. 1A). While we did not observe a significant difference in the ONL/INL ratio at the age of 3 months, the value was significantly decreased by 22% in 12-month-old *Acbd5*^*−/−*^ mice (*p* < 0.001), corroborating our published findings (Fig. 1B). Unexpectedly, however, the decreased ONL/INL ratio was not related to a significant decline in ONL thickness but a slight but significant increase in the INL (Fig. 1C, D). Likewise, the diameter of the OPL was significantly increased in 12-month-old *Acbd5*^*−/−*^ mice and that of the IPL in *Acbd5*^*−/−*^ mice of both ages (Fig. 1C, D).

In order to obtain more information on potential alterations in the numbers of distinct retinal cell types, confocal immunofluorescence microscopy (IF) was performed. Photoreceptor outer segments (POS) require permanent regeneration of their rhodopsin-bearing membrane discs, which could be imbalanced in a disorder with a disturbed FA metabolism. Therefore, we stained for rhodopsin to assess POS integrity but did not observe significant height differences between 3 nor 12-month-old *Acbd5*^*+/+*^ and *Acbd5*^*−/−*^ mice implying that membrane lipid renewal is still functional in photoreceptor cells (Fig. 1E, F). OCTs and ERGs from human RDLKD patients suggest a potential stronger impact on cone photoreceptor cells [17]. Immunostaining for cone arrestin was therefore used to examine a potential degeneration of cone photoreceptor cells. No significant decline was found among the 3-month-old animals, however, 12-month-old *Acbd5*^*−/−*^ specimens exhibited a significant 40% reduction in cone photoreceptor cell numbers (*p* < 0.01) (Fig. 1G, H). In C57BL/6J mice, cones comprise between 2 and 3% of the total photoreceptor cell number rather constantly across the whole retina surface [36, 37]. Hence, the reduced cone cell numbers are too low to significantly affect the net thickness of the ONL in *Acbd5*^*−/−*^ mice. Bipolar cells visualized by PKCα-staining transmit and integrate the photoreceptor primary information to ganglion cells (NeuN) forming the optic nerve with their axons. While 3-month-old *Acbd5*^*−/−*^ mice showed no significant alterations, a significant reduction of 28.6% and 40.0% (*p* < 0.05) in bipolar and ganglion cells was observed at the age of 12 months, respectively, implying that both retinal information processing cell types are compromised by metabolic alterations caused by the absence of ACBD5 (Fig. 1I-L). Since the general increase in the thickness of the inner retinal layers is not in line with the observed decline in major neuronal cell types, alternative cytological alterations have to explain these incongruent results. Neuroinflammatory processes can lead to structural rearrangements and invasion of the retina by glial cells. For example, INL thickening has been associated with neuroinflammatory optic neuritis in multiple sclerosis [38, 39], raising the question whether neuroinflammation may also account for the observed layer thickening in *Acbd5*^*−/−*^ mice. Hence, we extended our analysis by assessing morphology and state of the different retinal glia cells.

### Retinae of *Acbd5*^*−/−*^ mice exhibit an early activation of micro- and astroglia

**Fig. 2:**
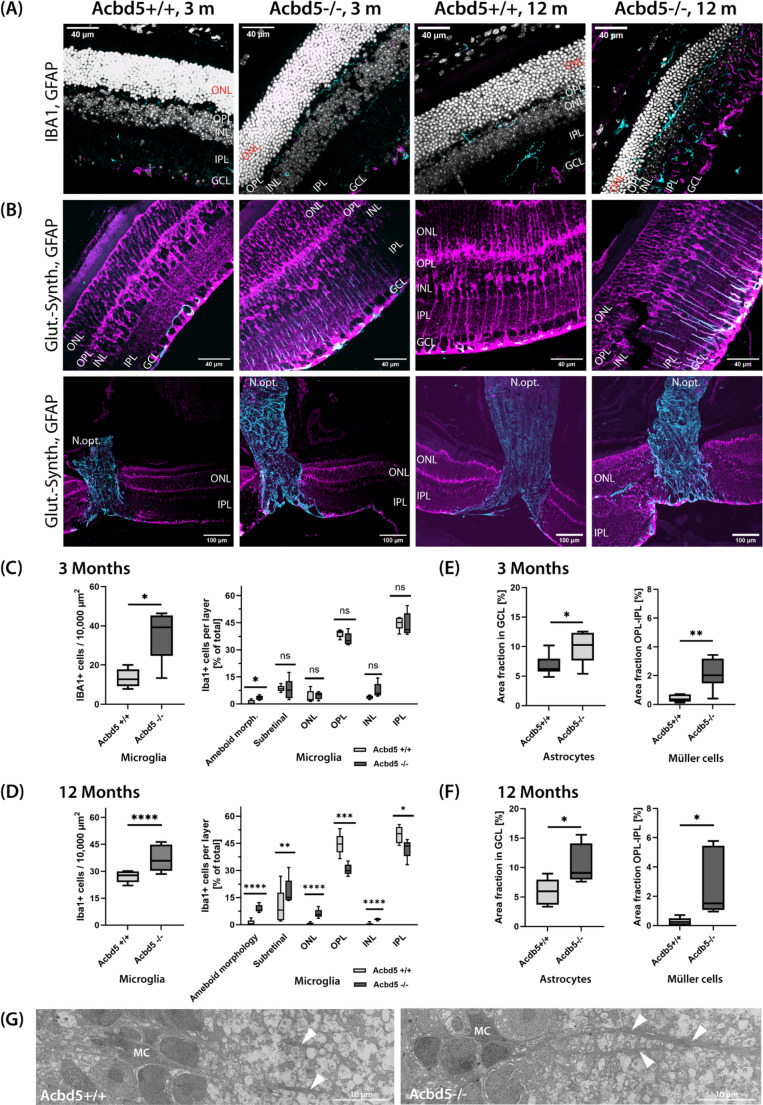
Neuroinflammation in the retina of *Acbd5*^*−/−*^ mice. (**A**) Representative IF images showing location and activation of microglia (IBA1, cyan), Müller cells and astrocytes (GFAP, magenta) in the retina of *Acbd5*^*−/−*^ mice. (**B**) Colocalisation of Glut-Synth. (magenta) and GFAP (cyan) in radial Müller cell projections in the IPL of the *Acbd5*^*−/−*^ mice. (**C**) Density of IBA1-positive microglia in all retinal layers and percentage of microglial cells in individual retinal layers (*n* = 5/genotype) from 3-month-old specimens. (**D**) Density of IBA1-positive microglia in all retinal layers and percentage of microglial cells in individual retinal layers (*n* = 5/genotype) from 12-month-old specimens. (**E**) Density of GFAP-positive astrocytes and Müller cells in the IPL, GCL and nerve fiber layer of the retina from 3-month-old mice. (**F**) Density of GFAP-positive astrocytes and Müller cells (MC) in the IPL, GCL and nerve fiber layer of the retina from 12-month-old mice. (**G**) Representative images of Müller cells projections into the IPL. Abbr.: ONL – outer nuclear layer, OPL – outer plexiform layer, IPL – inner plexiform layer, INL – inner nuclear layer, GCL – ganglion cell layer, N.opt. – Nervus opticus, Glut.Synth – glutamine synthetase; ns – not significant, * *p* < 0.05, ** *p* < 0.01, *** *p* < 0.001, **** *p* < 0.0001

Neuroinflammation is a typical hallmark in retinal dystrophies and may contribute significantly to the development of retinal pathologies [40]. To assess the potential activation of microglia, astrocytes and Müller cells, equatorial retina sections of 3-month- and 12-month-old *Acbd5*^*+/+*^ and *Acbd5*^*−/−*^ mice were stained for IBA1, GFAP and glutamine synthetase (Fig. 2A). The number of IBA1-positive cells was significantly increased by factor of 1.75 in *Acbd5*^*−/−*^ retinae of both ages (Fig. 2B, C). However, transition from the ramified into the activated amoeboid morphology was more prevalent in the older *Acbd5*^*−/−*^ cohort being 8.7-fold (*p* < 0.01) and 13.6-fold (*p* < 0.01) more frequent than in the respective control groups (Fig. 2B, C). Notably, local quantification in the different retinal layers revealed a migration of microglia from the inner into the outer retinal layers and subretinal space (Fig. 2C, D), which is a phenomenon also described in other cone/rod retinal dystrophies [40]. Documenting the phenotype progression, the relocalization of amoeboid microglia was more prevalent at an age of 12 month than 3 months, in line with the numerical increase in amoeboid morphology. Quantification of the GFAP IF signals in 3-month-old and 12-month-old mice revealed a significant activation of astroglia in the retinal nerve fiber layer (RNFL; 1.45-fold, 1.82-fold increase in percentage area in GCL/RNFL) extending into the optic nerve (Fig. 2A-C). In parallel, radial, retinal Müller cells showed also a markedly increased GFAP expression (5.24-fold, 11.16-fold elevated percentage area in OPL-IPL), suggesting that retinal neuroinflammation maybe a hallmark in the pathogenesis of the ACBD5-deficiency (Fig. 2D, E). Notably, conspicuous, thick and filament-rich Müller cell processes extending into IPL of *Acbd5*^*−/−*^ retina were regularly observed by EM analysis confirming the findings from the IF at the ultrastructural level (Fig. 2G). A summary of morphologic data from *Acdb5*^*−/−*^ mice points to a moderate retinal dystrophy, which is less characterized by major degeneration of neural retinal cells but more prominently by a conspicuous inflammation of the tissue. Based on this data, it is essential to obtain electrophysiological information to gain more data on potential functional consequences of the *Acbd5*^*−/−*^ phenotype.

### Full-field ERG implies pathologic functional changes in photoreceptor and INL cells

**Fig. 3:**
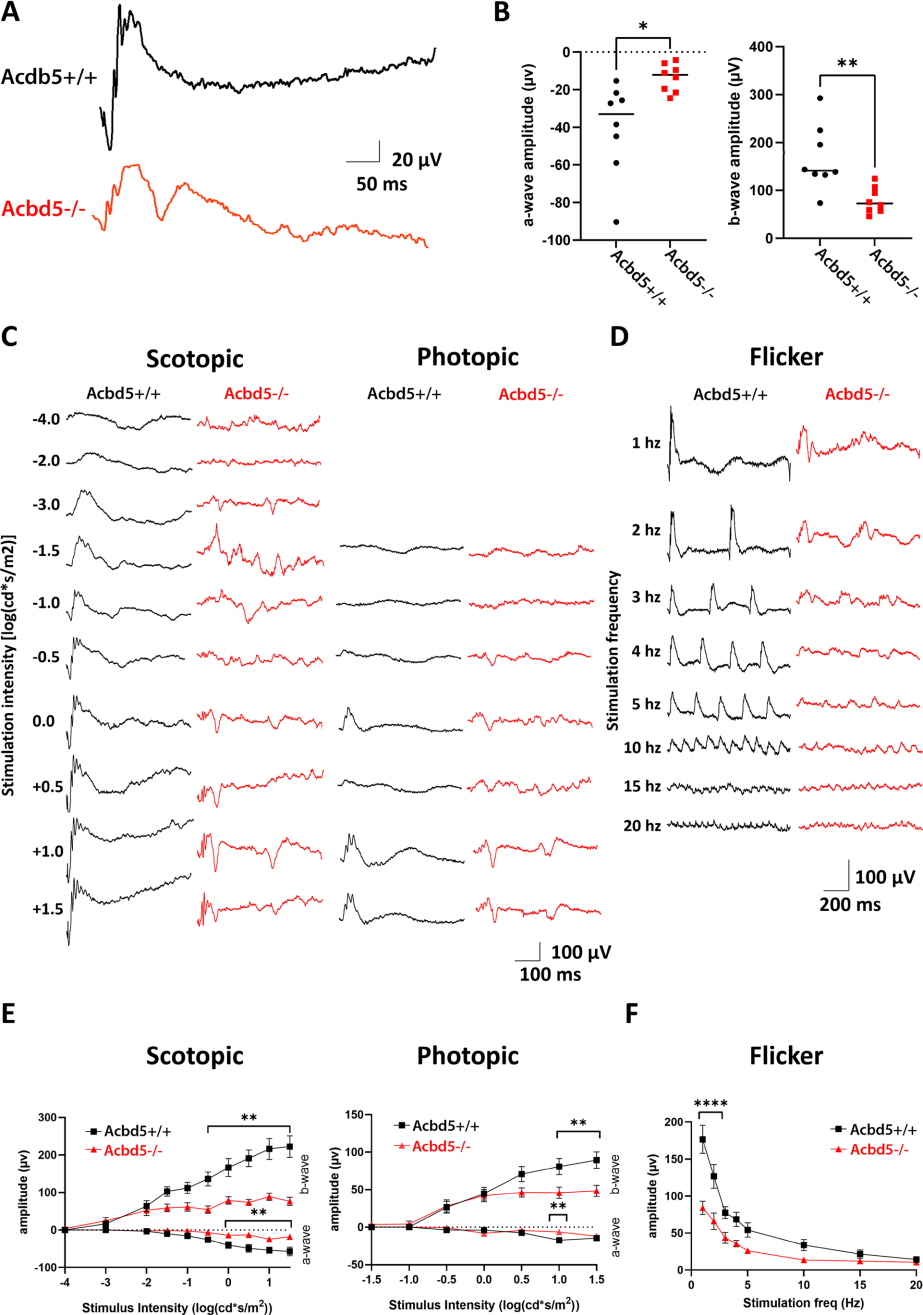
Full-field ERG characterization of *Acbd5*^*+/+*^ and *Acbd5*^*−/−*^. (**A**) Average scotopic ERG traces following flash stimulation at 0 log(cd*s/m^2^) from either *Acbd5*^*+/+*^ and *Acbd5*^*−/−*^. (**B**) Quantification of a-wave and b-wave amplitudes elicited following flash stimulation at 0 log(cd*s/m^2^). (**C**) Representative ERGs from single-flash flash stimulation sequences from − 4 to 1.5 log(cd*s/m^2^) for scotopic (dark-adapted) conditions, and − 1.5 to 1.5 log(Cd*s/m^2^) for photopic (light-adapted) conditions. (**D**) Representative flicker flash sequences with increasing stimulation frequency from from 1 to 20 hz. (**E**) Quantification of a- and b-waves as indicated, following scotopic and photopic flash stimulation sequences as shown in (C). (**F**) Quantification of b-waves following flicker ERG stimulation sequence as shown in (**D**). For all traces, black indicates *Acbd5*^*+/+*^ and red indicates *Acbd5*^*−/−*^ mice. Statistical analyses performed with (**B**) student’s unpaired t-test, and (**E**, **F**) two-way ANOVA with Sidak’s post-hoc test. * *p* < 0.05, ** *p* < 0.01, *** *p* < 0.001, **** *p* < 0.0001

To assess the light signal transmission across the different retinal layers of cone and rod pathways, ffERG was performed under scotopic and photopic conditions. Under scotopic conditions a-wave amplitudes were significantly reduced in the series of flash light stimulation (*p* < 0.05), suggesting that despite the normal morphological status of the ONL, rod photoreceptor signaling is functionally compromised in *Acbd5*^*−/−*^ retinae (Fig. 3A-C, E). Amplitudes of scotopic b-waves show an even more pronounced reduction below 50% of the control values (*p* < 0.01) suggesting that this difference does not only reflect the loss in rod photoreceptor outputs but also a dysregulation in bipolar cell signal transmission. Under photopic conditions, a-waves exhibited generally only low amplitudes thus impeding assessment of differences between *Acbd5*^*−/−*^ and *Acbd5*^*+/+*^ mice. However, the significant amplitude reduction at a stimulus intensity of 1.0 log(cd*s/m^2^) support the morphological findings of a reduced cone cell density (Fig. 3E). Likewise, the highly reduced b-wave amplitudes under photopic conditions underline a general dysfunction in signal transmission in the cone pathway. Flicker recordings of the b-wave also showed an irregular pattern at all measured frequencies suggesting a general disruption of the light signal pathways (3D). Remarkably, both scotopic as well as photopic ffERG recordings show reproducible, sharp secondary positive deflections in the resolving b-wave. The reason behind these secondary waves is currently unclear but might reflect pathologic alterations in the inner retina e.g. a slower recovery of bipolar cells, disturbed bipolar cell-amacrine cell interactions or a Müller cell dysfunction compromising K^+^ buffering.

To validate the differences in retinal thickness in *Acbd5*^*−/−*^ mice observed in fixed tissue sections in vivo, OCT was performed to measure the total retinal thickness (TRT) from the RNLF to the RPE. In contrast to the measurements from the retina sections, the OCT results demonstrated a slight but significant (*p* < 0.01) reduction of 10 μm in TRT in 12-month-old *Acbd5*^*−/−*^ mice compared to the age-matched *Acbd5*^*+/+*^ controls (Fig. S1A, B). Measurement of selected individual retinal layers in the OCT revealed a significant decrease in the ONL (*p* < 0.05), pointing to a limited degeneration of photoreceptor cells, while no significant alterations between *Acbd5*^*+/+*^ and *Acbd5*^*−/−*^ mice were observed for the IPL and INL. The conspicuously altered b-wave profiles observed by ffERG point electrophysiological alterations in the inner retinal layers. In this context, the discrepancy in layer thickness between fixed-tissue and the in vivo situation might be due to an altered ion homeostasis in the *Acbd5*^*−/−*^retina, which could result in differences in tissue water uptake during the embedding procedure.

### ACBD5 is most strongly expressed in retinal photoreceptor cells matching most closely with the localization pattern for the peroxisomal membrane protein PEX14

In order to correlate the morphological and electrophysiological alterations observed in the *Acbd5*^*−/−*^ mice with the distribution of peroxisomes and the expression of ACBD5 among the distinct retinal cell types, further IF analyses using selected peroxisomal proteins were performed. As reflected by the immunosignal for PEX14, which is required for the import of peroxisomal matrix proteins, peroxisomes are widely distributed across the retina showing highest abundancies in the RPE, PIS of photoreceptor cells and a subset of ganglion cells of *Acbd5*^*+/+*^ mice (Fig. S2A). Interestingly, colocalization with cone arrestin indicates that the PIS of cone cells contain higher peroxisome densities than rod cells (Fig. S2C). As already published previously [41], peroxisomal FA transporters and many enzymes exhibit in the retina a different distribution than the peroxin PEX14, which might be regarded as a peroxisomal housekeeping protein: catalase is most highly expressed in the RPE, while the peroxisomal ABC transporter ABCD3 exhibits a staining pattern, which best matches the signal for glutamine synthetase suggesting that Müller cells might show highest retinal peroxisomal β-oxidation activities (Fig. S2A). Remarkably, ACBD5-positive puncta show highest frequencies in the PIS matching most closely with the distribution of PEX14 but less with the peroxisomal acyl-CoA transporter ABCD3 (Fig. S2B, C). With respect to the postulated contribution of ACBD5 in VLCFA β-oxidation [18, 19], such a distribution pattern is intriguing and might underline a primary function of ACBD5 as a tethering protein, which is potentially not exclusively linked to peroxisomal FA metabolism. Importantly, no specific fluorescence signals for ACBD5 could be observed in *Acbd5*^*−/−*^ retinae, corroborating the efficiency of the *Acbd5* gene expression block in the mouse line (Fig. S2B, C). Hepatocytes of *Acbd5*^*−/−*^ mice exhibit a presumably compensatory peroxisome proliferation [23] Of note, no increases in the abundance of peroxisome were obvious in the *Acbd5*^*−/−*^ retina.

### The RPE of *Acbd5*^*−/−*^ mice shows signs of reduced membrane lipid catabolism and basal metabolite transport

**Fig. 4:**
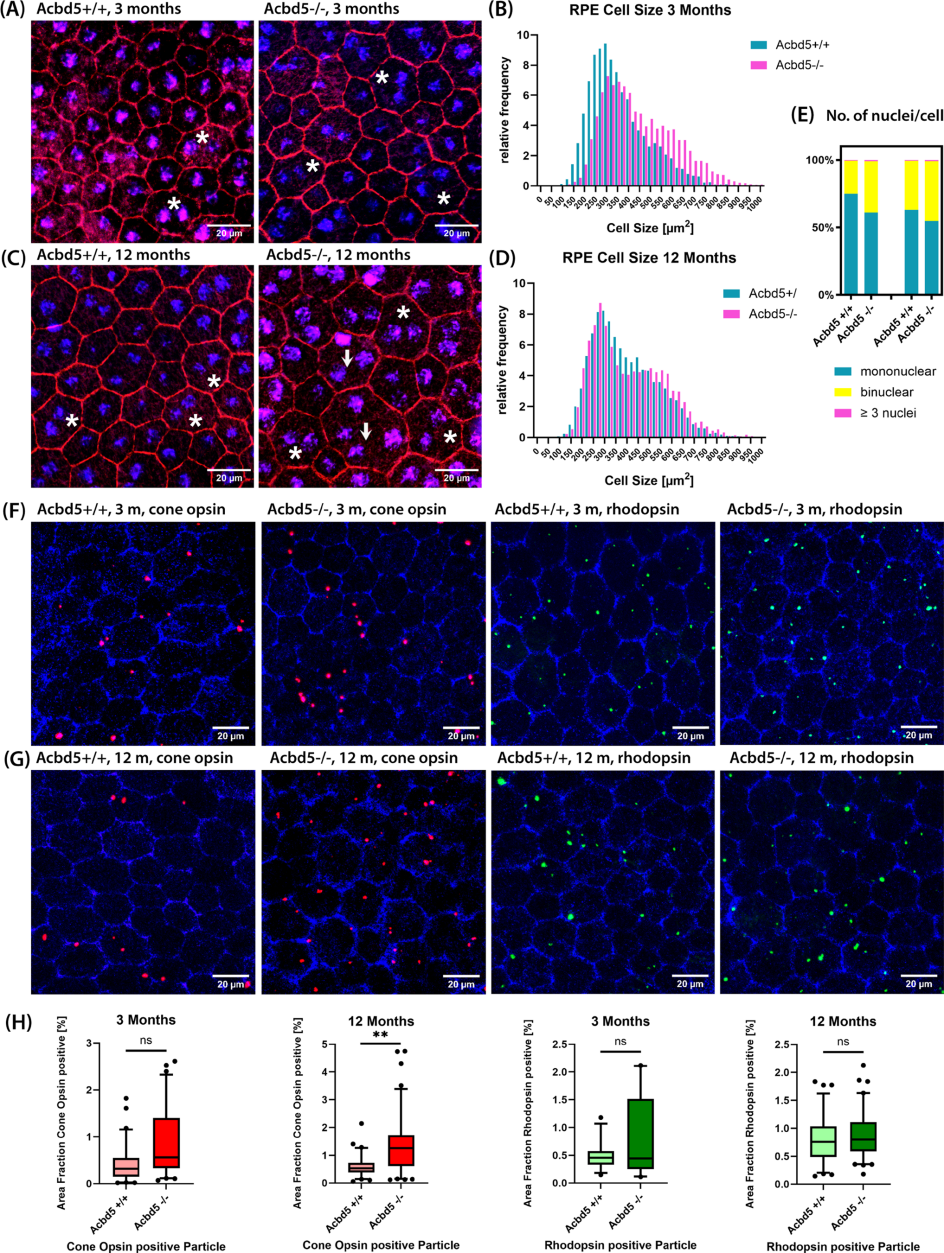
Morphology of the retinal pigment epithelium (RPE) in retina flat-mount preparations from 3-month and 12-month-old *Acbd5*^*−/−*^ and *Acbd5*^*+/+*^ mice (*n* = 10/genotype). (**A**) Representative IF images from 3-month-old specimens; cell borders are visualized by phalloidin (red), nuclei by TOPRO (blue); stars highlight binucleated, arrows multinucleated cells (> 2 nuclei). (**B**) Relative frequency distribution of the RPE cell size of 3-month-old mice. (**C**) Representative IF images from 12-month-old specimens; colors and labels as in (A). (**D**) Frequency distribution of the RPE cell size of 12-month-old mice. (**E**) Percentage of mono-/bi-/multinuclear cells in the RPE of 3- and 12-month-old *Acbd5*^*−/−*^ and *Acbd5*^*+/+*^ mice. (**F**, **G**) IF images showing cone opsin (red) and rhodopsin (green) positive vesicles in RPE flat mounts, cells borders are highlighted by peroxisomes (antibody against PEX14, blue) positioned in close proximity to the cell membrane. (**H**) Quantification of cone opsin and rhodopsin vesicles in RPE flat mounts (3 m: *Acbd5*^+/+^*n* = 6, *Acbd5*^−/−^*n* = 6; 12 m: *Acbd5*^+/+^*n* = 6, *Acbd5*^−/−^*n* = 7). ns – not significant, * *p* < 0.05, ** *p* < 0.01, *** *p* < 0.001

RPE cells continuously phagocytize and degrade exhausted distal ends of POS [42]. Accordingly, RPE cells have to degrade VLCFA contained in considerable concentrations in POS membrane lipids. Compromised peroxisomal β-oxidation in *Acbd5*^*−/−*^ mice could therefore ultimately lead to lysosomal lipid membrane accumulation inducing RPE cell degeneration [43]. As RPE cells are terminally differentiated, a decline in cell numbers is compensated by enlargement of neighboring cells. Flat mount RPE preparations were stained with phalloidin-red and TOPRO to detect potential changes in RPE cell size, shape and nucleus number (Fig. 4A, C). Indeed, 3-month-old *Acbd5*^*−/−*^ mice showed a significant shift towards larger bi- or multinucleated cells (Fig. 4B, E, Fig. S3A, B), which was even more pronounced in 12-month-old *Acbd5*^*−/−*^ mice (Fig. 4D, E). However, 12-month-old *Acbd5*^*+/+*^ controls also showed signs of RPE degeneration resulting in insignificant differences in average cell size and percentage of mononucleated cells between both genotypes at this age. A dysfunctional membrane lipid catabolism might lead to compromised phagosomal degradation of POS membrane discs. Unexpectedly, quantification of rhodopsin and cone opsin-positive puncta in RPE flat-mount preparations from 12-month-old *Acbd5*^*−/−*^ mice only revealed a significant increase by a factor of 2.3 in phagosomes containing membrane discs from cone POS (Fig. 4F-H). This is an unexpected finding, as VLC-PUFA were reported to be specifically required for rod photoreceptor function [44], implying that the degradation of POS membranes would be preferentially compromised in this cell type. Hence, other lipid metabolites impeding lipid membrane degradation might be altered in cone POS of *Acbd5*^*−/−*^ mice. After export from lysosomes, FA cleaved from phospholipids will be, depending on their chain length, shuttled to mitochondria or peroxisomes for further degradation. In case of a compromised breakdown, they will be deposited as triglycerides in lipid droplets (LD).

**Fig. 5:**
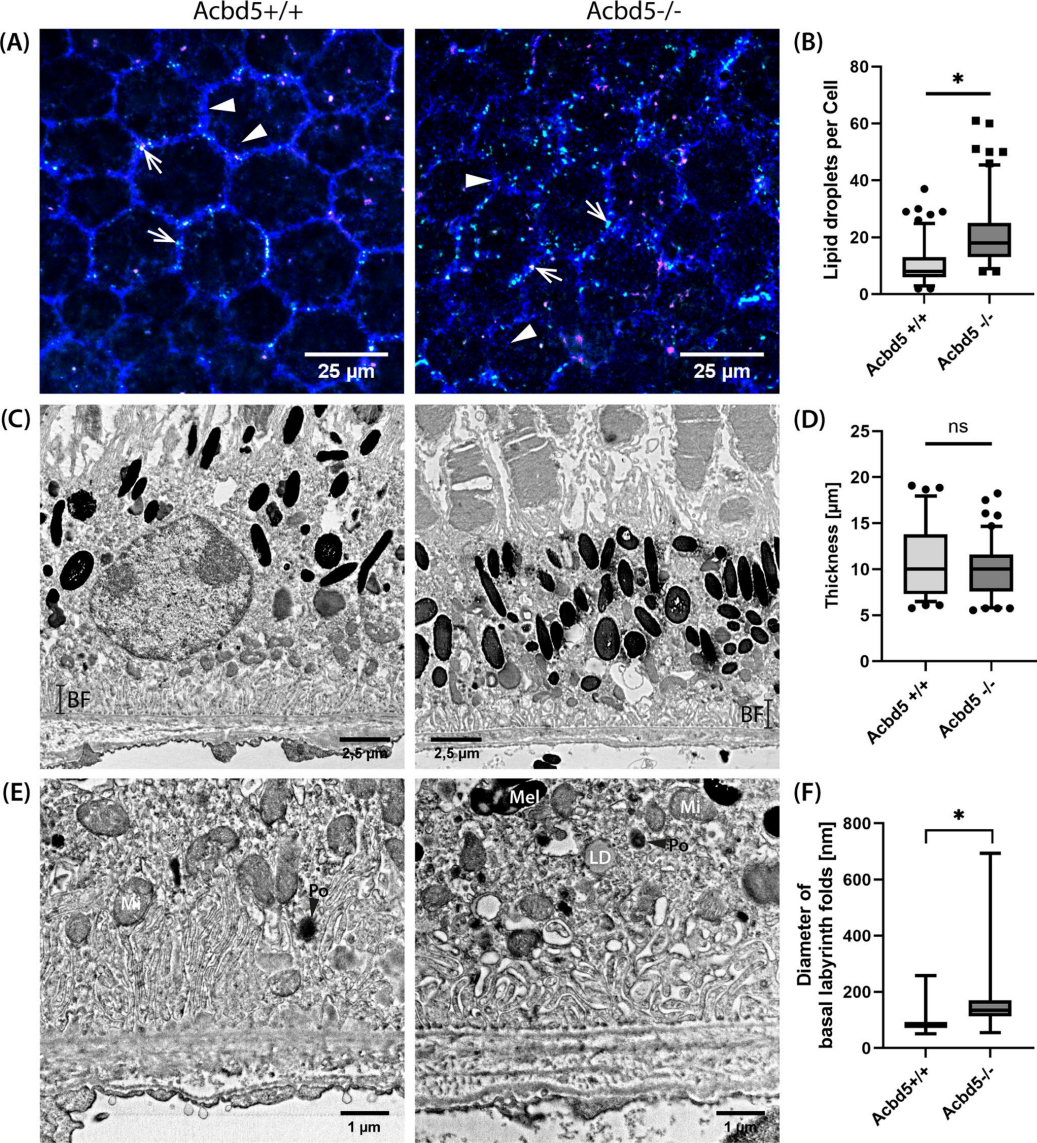
Subcellular organization of RPE cells from 12-month-old *Acbd5*^*−/−*^ and *Acbd5*^*+/+*^. (**A**) Nile-Red-stained RPE flat-mount preparations showing 488 nm-exited fluorescence for neutral lipids (cyan) and 561 nm-exited fluorescence for phospholipids (magenta). Note that neutral lipids show residual fluorescence at 561 nm resulting partially in white spots in overlays; additionally, peroxisomes were identified by PEX14 antibodies (shown in blue). Examples for singular and accumulations of peroxisomes are indicated by arrowheads, lipid droplets are indicated by arrows. (**B**) Quantification of cellular lipid droplets marked in cyan in (A) (*n* = 5 per genotype). (**C**) EM images giving an overview of RPE cell morphology (*Acbd5*^*+/+*^*n* = 3, *Acbd5*^*−/−*^*n* = 4). (**D**) Quantification of the average height of the RPE cells from the Bruch membrane to the POS (nuclear regions excluded). (**E**) Magnifications from the deeply folded basal RPE cell membrane including the Bruch membrane. Abbr.: Mel -melanosome, Mi – mitochondrion, PO – peroxisome, BF – basal folds, Note, that the folds are less frequent and rather coarse in the *Acbd5*^*−/−*^ RPE (**F**) Quantification of the diameter of individual folds in the basal cell membrane (*Acbd5*^*+/+*^*n* = 3, *Acbd5*^*−/−*^*n* = 4). ns – not significant, * *p* < 0.05, ** *p* < 0.01, *** *p* < 0.001

To evaluate changes in LD content, flat-mount RPE preparations were stained by Nile Red, which shifts its emission maximum towards shorter wave lengths in more hydrophobic solutes [45] and hence allows discriminating phospholipids from neutral lipids. (Fig. 5A, B). In the RPE of *Acbd5*^*−/−*^ mice LD exhibited more than twice the amount than in *Acbd5*^*+/+*^ mice (*p* < 0.05). Moreover, while LD were preferentially found at the periphery of the *Acbd5*^*+/+*^ RPE cells, they increasingly located in the RPE’s inner cytoplasm in *Acbd5*^*−/−*^ mice (Fig. 5A, B), which points to a dysfunction in peroxisomal FA degradation possibly impeding effective POS degradation. Since we observed a significant peroxisome proliferation in hepatocytes of *Acbd5*^*−/−*^ mice [23] and RPE cells like hepatocytes express PPARα; Pex14 IF was used to evaluate potential compensatory peroxisome proliferation. However, no evidence for a peroxisome proliferation were found in the RPE cells.

In order to evaluate, if the alterations in lipid metabolism may lead to detrimental changes in the RPE cellular morphology, transverse eye sections from *Acbd5*^*−/−*^ (*n* = 4) and *Acbd5*^*+/+*^ (*n* = 3) mice were analyzed by EM. RPE cells presented as a single, flat layer with a typical polar cell morphology with long and slender microvilli projecting from the apical cell membrane to surround the POS. Deep, slender folds enlarge the basolateral membrane in order to facilitate efficient metabolite exchange with underlying choroidal blood vessels across the Bruch’s membrane. While the apical two thirds of the cytosol contain numerous phagosomes, melanosomes and lysosomes, mitochondria are preferentially located next to the basal folds providing ATP required for active transport processes (Fig. 5C). Small peroxisomes, visualized by alkaline DAB staining [46], were found adjacent to the basolateral cell membrane, as already shown by IF (Fig. S3C). No gross ultrastructural alterations were observed in the RPE of *Acbd5*^*−/−*^ mice. Likewise, the average RPE height was with 9.8 μm comparable to 10.6 μm in *Acbd5*^+/+^ controls (Fig. 5D). Apical microvilli projected in comparably thin and long microvilli from the apical cell membrane as also confirmed in 3D-reconstructions from Nile Red-stained confocal imaging stacks from RPE flat-mount preparation (Fig. S3D). However, an obvious difference between *Acbd5*^*−/−*^ and *Acbd5*^*+/+*^ RPE cells was found in basal fold morphologies: these were less slender and less densely stacked in *Acbd5*^−/−^ mice (Fig. 3E). Quantification of the fold diameter confirmed this visual impression demonstrating a significant difference in average values of 84.4 nm for *Acbd5*^*+/+*^ and 149.5 nm for *Acbd5*^*−/−*^ mice (Fig. 3F). Moreover, in the *Acbd5*^*−/−*^ RPE, peroxisomes were frequently found attached to LDs possibly to compensate for reduced VLCFA import capacities [19]. Taken together, these results suggest a reduced RPE capacity to degrade membrane lipids and therein contained VLCFA. This might lead to accumulation of VLCFA in the RPEs own biomembranes leading to a dysfunctional membrane organization thereby initiating structural changes in the RPE’s basal folds which could then reduce metabolic exchange with the choroidea. Interestingly, such a disorganization of the RPE basal labyrinth has been previously described in the ageing or degenerating retina and was associated with reduced transport capacities between the RPE and choroidal blood vessels [47, 48].

### Lipidomics analysis reveals an accumulation of VLC-PUFAs in retinal phosphatidylcholines

**Fig. 6:**
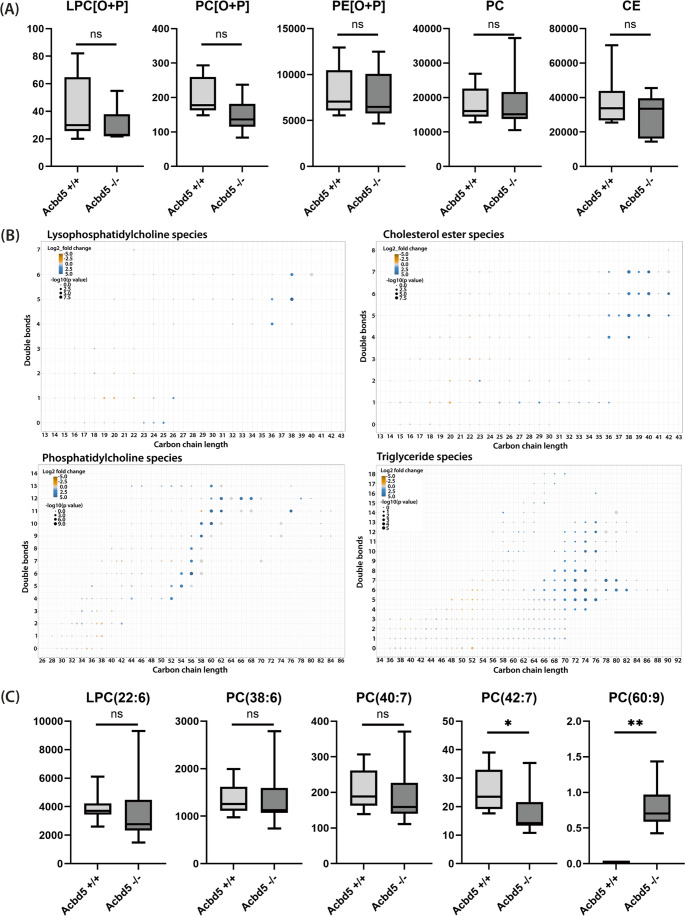
Lipid composition of total retina homogenates from *Acbd5*^*−/−*^ and *Acbd5*^*+/+*^ mice (*n* = 8 per genotype) determined by ESI-MS. (**A**) Comparison of the amounts of distinct ether/ester lipid classes: LPC [O + P] – alkyl-/alkenyl-lysophosphatidylcholines, PC [O + P] - alkyl-/alkenyl-phosphatidylcholines, PE [O + P] - PC [O + P] - alkyl-/alkenyl-phosphatidylethanolamines, PC – phosphatidylcholines, CE – cholesteryl esters. (**B**) Comparison of the fatty acid (FA) composition of selected lipid classes, circles in blue show lipid species elevated and circles in orange lipid species decreased in *Acbd5*^*−/−*^ retinae, the circle size signifies the measure of significance of the alteration, the x-axis depicts the FA carbon chain length, the y-axis the number of double bonds/FA. (**C**) Direct comparison of selected (lyso-)phosphatidylcholines ((L-)PC) representative for the following phospholipid FA combinations: LPC(22:6): DHA, PC(38:6): DHA + palmitic acid, PC(40:7): DHA + oleic acid, PC(42:7) DHA + eicosenoic acid, PC(60:9); DHA + C38:3. ns – not significant, * *p* < 0.05, ** *p* < 0.01

In order to correlate the retinal pathology with metabolic changes caused by disruption of peroxisome functions, lipids were extracted from homogenates from the neural retina of *Acbd5*^*−/−*^ and *Acbd5*^*+/+*^ mice aged 12 months (*n* = 8 per genotype) and analyzed by MS [35]. The loss of ACBD5-mediated membrane contact sites between the ER and peroxisomes might result in reduced ether lipid synthesis capacities since the pathway intermediate alkyl-DHAP has to be transferred from peroxisomes to the ER to complete the pathway [2]. However, the lipidomics analysis did not reveal any significant alterations at the level of lipid classes (Fig. 6A).

As ACBD5-deficient cells show reduced VLCFA β-oxidation capacities, we compared the individual lipid classes for quantitative changes in carbon chain length (Fig. 6B). Values for lysophospholipids give a reliable estimate for the amount of individual FA in glycerophospholipids. While lysophosphatidylethanolamines (LPE), lysophosphatidylserines and lysophosphatidic acids did not exhibit apparent changes in their FA spectrum, lysophosphatidylcholines (LPC) showed a significant accumulation of VLC-PUFA with 36–40 carbon atoms (Fig. 6B, Fig. S4). A comparable significant VLC-PUFA accumulation was observed in phosphatidylcholines (PC), triglycerides (TG) and cholesterolesters but not in phosphatidylethanolamines, phosphatidylserines, and phosphatidic acids (Fig. 5B, Fig. S4, S5 and Suppl. Data 1) implying that hyper-elongated FA are specifically incorporated into PC and TG. Notably, VLCFA accumulation was not observed in any ether phospholipid class.

DHA is a ω3-PUFA found in unusually high amounts in retinal phospholipids and is essential for a healthy retina [26]. Moreover, it requires peroxisomal β-oxidation for its synthesis. Initial FA elongation from α-linolenic acid requires FA elongases (ELOVL) and desaturases localized at the ER to produce tetracosahexaenoic acid (TPA, C24:6n-3), which is finally imported into peroxisomes for a single chain-shortening step producing DHA (C22:6n-3). Hence, intermediates have to be shuttled between the two organelles potentially decelerating DHA synthesis in *Acbd5*^*−/−*^ mice, in which PO-ER membrane contacts are reduced [23]. However, C22:6 was neither reduced in LPC nor LPE of the *Acdb5*^*−/−*^ retinae (Fig. 6C, Suppl. data 1). Likewise, the abundant PC species PC(38:6) (22:6 + 16:0), PC(40:7) (22:6 + 18:1) containing with a high probability DHA as one FA showed comparable quantities. Only PC(42:7) likely containing significant amounts of PC combining C24:6 + C18:1, was slightly reduced in *Acbd5*^*−/−*^ retinae. These findings might imply that DHA intermediates accumulating at the ER are “over”-elongated to VLC-PUFA (see PC(60:9) in Fig. 5C and Fig. S4). Since DHA for retinal phospholipids might be synthesized in the liver and supplied via the blood stream, free DHA plasma concentrations were determined. In both 3-month-old and 12-month-old *Acbd5*^*−/−*^ mice, DHA concentrations were approximately 70% of the respective values for *Acbd5*^*+/+*^ mice (Fig. S6, 3 m: *p* < 0.01 and 12 m: *p* < 0.05) revealing likewise only very moderate differences between both genotypes.

Interestingly, a significant alteration in FA composition was also observed for cardiolipins (Fig. S6). While PC exhibited an increase towards VLC-PUFA, this trend to elongated FA is less prominent in the mitochondrial cardiolipins of *Acbd5*^*−/−*^ mice. However, cardiolipins generally showed a higher degree of unsaturation. Remarkably, cardiolipin remodeling towards a higher content of unsaturated FA has been reported to alter OXPHOS packaging in the inner mitochondrial membrane [49]. As a consequence, the disturbed peroxisomal lipid metabolism in *Acbd5*^*−/−*^ mice could have a direct impact on mitochondrial energy homeostasis.

### MALDI MS imaging reveals a significant enrichment in of VLC-PUFA in the plexiform layer of the ACBD5-deficient retina

**Fig. 7:**
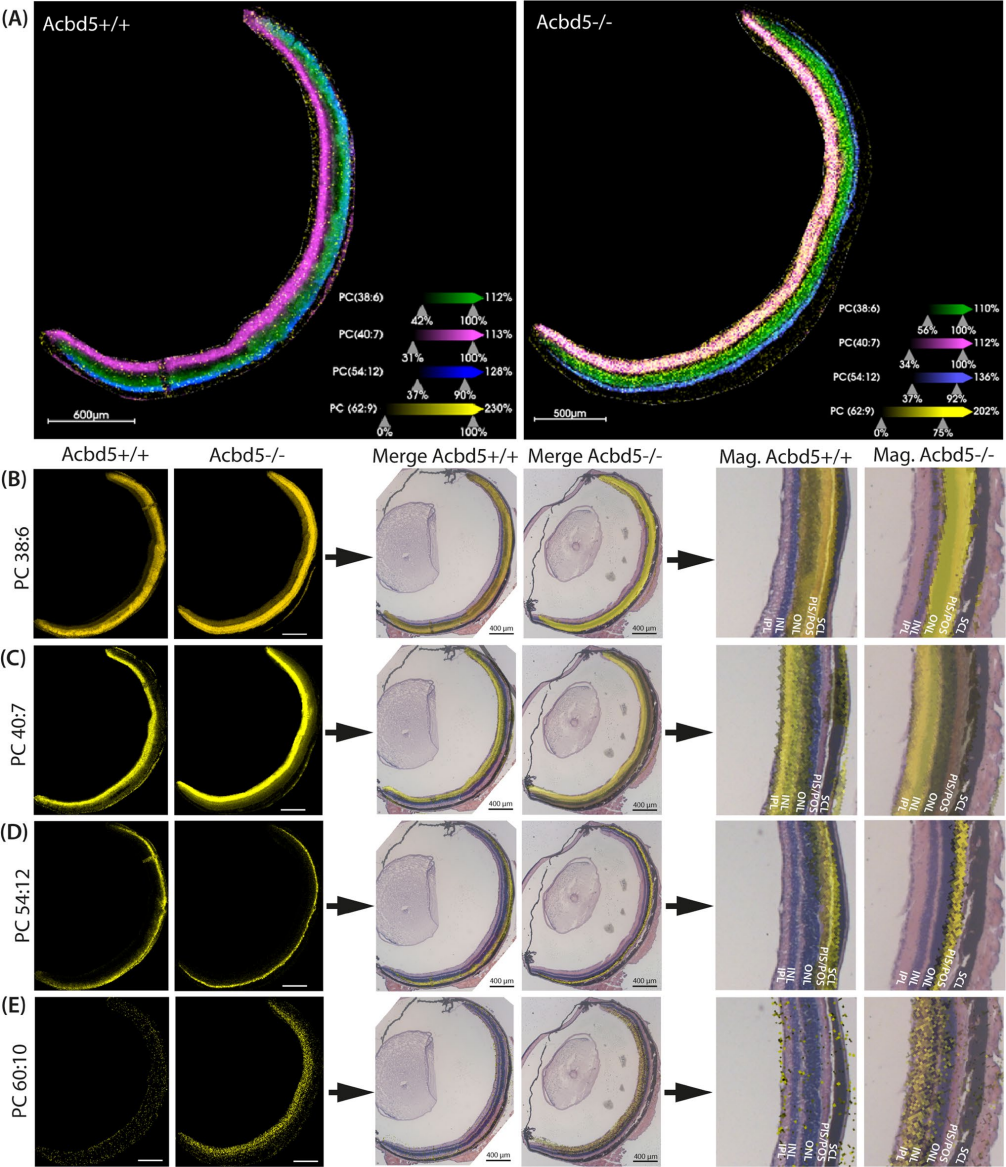
Distribution of selected PC species in sagittal sections of *Acbd5*^*−/−*^ and *Acbd5*^*+/+*^ retinae determined by MALDI MS imaging (*n* = 5/genotype). (**A**) Overlay of m/z signal intensity distribution for PC(38:6) (green), PC(40:7) (magenta), PC(54:12) (blue), PC(62:9) (yellow). (**B** – **E**) Localization of the individual PC species among the retinal layers; to this end, distinct m/z signals representing PC(38:6) (*m/z* 828.55, [M + Na]^+^, mass error 1.45 ppm) (**B**), PC(40:7) (*m/z* 832.58, [M + H]^+^, mass error 3.0 ppm) (**C**), PC(54:12) (*m/z* 1040.71, [M + Na]^+^, mass error 1.06 ppm) (**D**), PC(60:10) (*m/z* 1106.85, [M + H]^+^, mass error 1.99 ppm) exemplary transverse retina sections were merged with the HE-stained consecutive section from the same specimen. Abbr.: SCL – sclera, PIS/POS – photoreceptor inner, outer segment, ONL – outer nuclear layer, INL – inner nuclear layer, IPL – inner plexiform layer

The lipidomics analysis of the total retina homogenates gives a general overview of the lipid changes occurring in *Acbd5*^*−/−*^ mice. The retina is, however, a highly stratified tissue, in which the numerous different cell types cooperate at different organizational levels. In this context, the different retina layers possess unique phospholipid compositions, which might be required to facilitate the specialized membrane protein functions in distinct cell types [50, 51]. Hence, in order to obtain information on potential local differences in the observed changes in PC FA composition, sagittal eye sections from 12-month-old *Acbd5*^*−/−*^ and *Acdb5*^*+/+*^ mice (*n* = 5) were subjected to MALDI MS Imaging [34]. Notably, the membrane lipids from the different retina layers exhibit a unique FA composition reflecting specializations in the physical properties of the phospholipid membranes (Fig. 7A, Fig. S7).

Substantial DHA concentrations are especially crucial for POS integrity and maintenance [52]. The PC(38:6) and PC(40:7), PC(44:12) represent to a high degree combinations of DHA (C22:6) with palmitate (C16:0) oleate (C18:1), or two DHA chains, respectively, which are all unusually abundant FAs combinations in phospholipids of the retina [53]. Thus, in order to exclude that phospholipids containing DHA as one FA may be locally decreased in the retina of *Acbd5*^*−/−*^ mice, the quantitative distribution of PC(38:6), PC(40:7) and PC(44:12) were examined (Fig. 7B, C, Fig. S7A, B-D, J, Tab. S3). Remarkably, the three PC show a different spatial distribution, with a higher abundance of PC(38:6) in the region of the POS to the ONL, while PC40:7 shows higher concentrations in the layers between OPL and IPL and PC44:12 is most concentrated in the OPL region. For all three PC, however, no significant intensity difference was detected between *Acbd5*^*−/−*^ and *Acbd5*^*+/+*^ specimens (Tab S3). Likewise, PC(42:9) (suggested to contain C22:6 + C20:3) was not altered in *Acbd5*^*−/−*^retinae (Fig. S7C) overall indicating that the retinal DHA levels are also not locally decreased by the loss in ACBD5, which implies that the retinal pathology might not be ascribed to DHA shortages. Next, we compared signal intensities for PC, which most likely contain one VLCFA with a chain length between C26 and C36 (PC(54:12), PC(58:9), PC(60:9), PC(60:10), PC(62:10)). Remarkably, the different VLCFA-containing PC showed distinct distribution patterns among the retinal layers (Fig. 7D, E, Fig. S7E-J). PC(54:12) dominates in the PIS/POS region of the retina, thus confirming the high concentration of VLC-PUFA in photoreceptor PC [54]. Unexpectedly, however, its signal intensity did not differ between *Acbd5*^*−/−*^ mice and controls. Likewise, PC(62:10) showed strong and comparable signals in the outer (PIS/POS) region of *Acbd5*^*−/−*^ and *Acbd5*^*+/+*^ retinae. Additionally, however, PC(62:10) was specifically and highly enriched in the inner retinal layers from *Acbd5*^*−/−*^ mice (Fig. S7I, J, Tab. S3, *p* < 0.001). Notably, also PC(60:10) accumulated selectively in the inner region of the *Acbd5*^*−/−*^ retina ranging from the GCL or IPL to OPL (Fig. 6E, Fig. S5F, H, I, *p* > 0.001). Overlays of MSI-spectra revealed a corresponding spatial accumulation of PC(58:9) and PC(60:9) (Fig. S7J). Accordingly, PC with most highly unsaturated VLC-PUFA appear to play an important role in membranes of PIS/POS. Notably, however, their degradation is locally still functional. Different results were observed in the inner retinal layers dominated by bipolar, amacrine, Müller and ganglion cells. In these regions, VLCFA-containing PC are strikingly increased in *Acbd5*^*−/− *^mice. These findings are in line with the results for b-waves from the ffERG, which point to a prominent disruption of bipolar cell or Müller cell function. Hence, the pathologic alterations in the retina from *Acbd5*^*−/−*^ mice might initiate in cells from the plexiform layers, while the dysfunction of photoreceptor cells might be a secondary event triggered by a primary disruption of bipolar or Müller cell function.

### Ribbon synapses between photoreceptor cells and horizontal/bipolar cells show signs of degeneration in the ACBD5-deficient retina

**Fig. 8:**
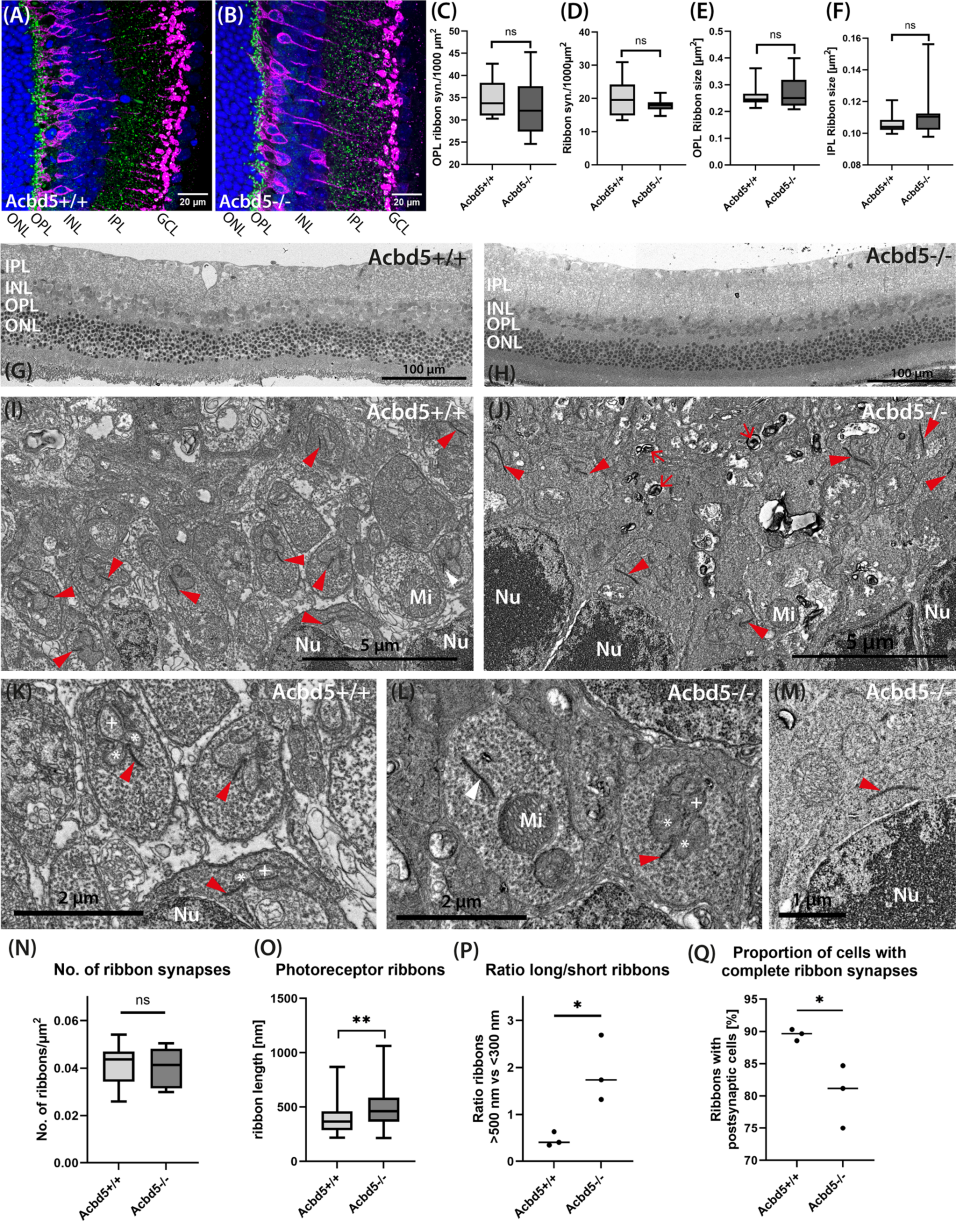
Ribeye IF and electron microscopy analysis of the retina from 12-month-old *Acbd5*^*−/−*^ and *Acbd5*^*+/+*^ mice. (**A**, **B**) Representative transverse retina sections from *Acbd5*^*+/+*^ and *Acbd5*^*−/−*^ mice (*n* = 5/genotype) depicting ribbon synapses in green (Ribeye IF) and bipolar cells in magenta (PKCα IF); nuclei are shown in blue (TOPRO). (**C**-**F**) Quantification of ribbon synapse size and density in the OPL and IPL of the IF images. (**G**, **H**) General view of cross sections through the retina from *Acbd5*^*+/+*^, *n* = 3 (**G**) and *Acbd5*^*−/−*^ mice, *n* = 3 (H); magnifications are derived from the OPL. (**I**, **J**) Overview of the OPL from *Acbd5*^*+/+*^ (**G**) and *Acbd5*^*−/−*^ (**H**) retinae, ribbon synapses are highlighted by red arrowheads. (**K**) Typical photoreceptor ribbon synapses from *Acbd5*^*+/+*^ retinae, the presynaptic ribbon is highlighted by red arrowheads, horizontal cell terminals by stars, bipolar cell projections by a ”plus” and lysosomes with residual inclusions by red arrows. (**L**) Representative ribbon synapses in the *Acbd5*^*−/−*^ OPL; extend ribbons without contact to postsynaptic structures as highlighted by the white arrowhead were more frequently observed in the ACBD5-deficient OPL. (**M**) Presynaptic ribbon retracted into the vicinity of the nucleus of a photoreceptor cell in an *Acbd5*^*−/−*^ retina. (**H**-**L**) Quantification of the ribbon synapse frequency and morphology analyzed by EM. Abbr.: Mi – mitochondrion, Nu – nucleus, IPL – inner plexiform layer, INL – inner nuclear layer, OPL – outer plexiform layer, ONL – outer nuclear layer; ns – not significant, * *p* < 0.05, ** *p* < 0.01

Bipolar cells span the inner and outer plexiform layer of the retina and could therefore be a direct or indirect target of the pathologic elevation of membrane VLC-PUFA observed by MALDI MS imaging (Fig. 8A, B). Likewise, physiological Müller cell K^+^ transport is a prerequisite for a functional synaptic communication between photoreceptor and bipolar cells. Ribbon synapses from the OPL are prominent structures, in which signals from photoreceptors are transmitted to a postsynaptic cluster of horizontal and bipolar cells, while those from the IPL connect bipolar cells of the cone pathway with ganglion cells [55]. Of note, a knockout of the ribbon-component RIBEYE results in a most prominent reduction in ffERG b-wave amplitudes [32, 56]. Hence, the strong reduction in b-wave amplitudes identified in *Acbd5*^*−/−*^ mice might in part point to disrupted photoreceptor outputs to bipolar cells. Moreover, synaptosomes from ribbon as well as conventional synapses of the OPL and IPL contain considerable amounts of VLC-PUFA, suggesting that VLC-PUFA-containing phospholipids play a role in synaptic function [57]. Confocal IF microscopy using RIBEYE antibody labeling was used to monitor potential changes in ribbon synapse distribution (Fig. 8A, B). However, neither in the OPL nor IPL significant changes in the density and size of RIBEYE-positive puncta were observed (Fig. 8C-F). At the ultrastructural level, the eponymous ribbons from the OPL are crescent-shaped electron dense protein filamentous structures positioned perpendicular to the presynaptic membrane, on which numerous synaptic vesicles are docked. In ultrathin sections, ribbons can be either cut in cross sections with a short ribbon and adjacent bipolar/horizontal projections or horizontal presenting a single long ribbon band (Fig. 8G, H). Visual comparison of EM images suggested that elongated ribbons occurred considerably more frequently in the OPL of *Acbd5*^*−/−*^ than *Acbd5*^*+/+*^ retinae (Fig. 8I-M). In order to validate this initial impression, we determined the length of 249 and 256 ribbons from 3 *Acbd5*^*−/−*^ and *Acbd5*^*+/+*^ mice, respectively and detected a significantly higher average ribbon length in the *Acbd5*^*−/−*^ OPL, while the overall frequency of ribbons was not altered (Fig. 8N, O). Retraction of postsynaptic processes from the presynaptic terminals has been described as a phenomenon in degenerating retinas [58]. In line, we observed a higher frequency of elongated ribbons, which were not associated with postsynaptic terminals in the *Acbd5*^*−/−*^ retinae (Fig. 8P, Q). Moreover, the OPL from *Acbd5*^*−/−*^ retinae regularly contained vesicular structures containing electron dense sheets, which might represent lysosomes enclosing undigested lipid membranes. IF staining against the lysosomal membrane protein LAMP1 confirmed the accumulation of small lysosomes in the OPL (Fig. S8). In summary, these changes in OPL from *Acbd5*^*−/−*^ retinae suggests degenerative processes in bipolar cells, leading to terminal dendrite retraction from ribbon synapses and accumulation of lipid membranes in their dendritic compartment.

## Discussion

The retina exhibits a highly specialized membrane lipid composition with an unusually high percentage of PUFA and VLCFA-containing phospholipids [27, 59]. Since peroxisomes are essential for VLCFA degradation, DHA- and plasmalogen synthesis, a dysfunctional peroxisomal metabolism will inevitably impact retinal lipid homeostasis. Accordingly, retinal degeneration is a common pathology in inherited peroxisomal disorders. Since the first ACBD5-deficient patient was identified in a genetic retinal dystrophy screen [11], its ocular pathology has received special attention and led to its OMIM name RDLKD. However, despite the common visual decline in RDLKD patients, information on the cellular and metabolic alterations in the patients’ retina is still sparse. Thus, in order to gain information on the retinal pathogenesis, we correlated retinal alterations at the cellular and subcellular level with lipidomics changes in an *Acbd5*^*−/−*^ mouse model.

According to light microscopic histologic results, the retinal degeneration in *Acbd5*^*−/−*^ mice is only moderate inducing no gross alterations in the retinal architecture. However, specific quantification of individual cell types and results from ffERGs revealed compromised photoreceptor, bipolar and ganglion cells. Degeneration of photoreceptor cells was also reported for the Gly357* mouse model for ACBD5-deficiency [24]. However, according to our ffERG recordings, the retinal dysfunction is especially prominent in cells from the INL. This correlates with the local accumulation of medium-unsaturated VLC-PUFA in the inner retinal layers (OPL, INL, IPL, GCL) and corresponds with the observed thickening of the same layers in HE-stained retina sections. In contrast, highly unsaturated VLC-PUFA found to be prevalent in the region of POS/PIS were unchanged, suggesting that cell-type alterations in membrane lipid composition of the *Acbd5*^*−/−*^ retina may trigger the pathogenesis.

Unlike *Acbd5*^*−/−*^ mice, peroxisomal multifunctional protein 2 (MFP2) knockout mice exhibit a complete block in peroxisomal β-oxidation. Accordingly, *Mfp2*^*−/−*^ mice develop a significantly more severe retinal pathology with a marked deterioration of the neural retina at early ages [7]. Notably, in contrast to *Acbd5*^*−/−*^ mice, the *Mfp2*^*−/−*^ mice showed a developmental phenotype reflected by significant POS shortening and a severe photoreceptor cell degradation at already 9 weeks of age correlating with a significant reduction in scotopic ERG a-waves. Hence, the rod photoreceptor cells appear to be most severely affected by the complete loss in peroxisomal FA β-oxidation. Lipid analysis of the whole retina showed in addition to an increase in VLC-PUFA, a marked decrease in DHA-containing PE and PC [7]. Hence, while both mouse lines resemble each other in the accumulation of VLC-PUFA in PC, only *Mfp2*^*−/−*^ mice exhibit a significant reduction in retinal DHA levels. Interestingly, *Crx*-*Mfp2*^*−/−*^ mice with a rod, cone and bipolar cell-specific *Mfp2* deletion showed changes in the lipid spectrum similar to the *Acbd5*^*−/−*^ mice: changes are similarly restricted to VLC-PUFAs found to be increased in TGA, CE and PC, while parameters for DHA levels remained unaltered [60]. Remarkably, *Cre*-*Mfp2*^*−/−*^ mice, which develop their phenotype considerably later than the *Mfp2*^*−/−*^ mice, do not present with a significant reduction in retinal thickness and detectable degeneration of photoreceptor cells but exhibit a significantly reduced bipolar cell numbers. Remarkably, *Cre-Mfp2*^*−/−*^mice also show changes in photoreceptor ribbon morphology, which appear to be similar to the alterations described in this manuscript but which where only assessed on a qualitative level. A comparable phenotype was as well reported for *Cre*-*Pex5*^*−/−*^ mice suggesting that this bipolar cell pathology is mainly caused by the disruption of peroxisomal β-oxidation [60]. Interestingly, the degeneration of photoreceptor cells in *Mfp2*^*−/−*^ and *Acox1*^*−/−*^ mice was significantly ameliorated by nutritional DHA supplementation [10, 61]. An RPE-specific knockout of *Mfp2* could further show that the photoreceptor degeneration is primarily caused by the loss in peroxisomal β-oxidation in the RPE preventing normal membrane lipid degradation of the POS [43].

Interestingly, Pex1-G844D mutant mice, which as *Acbd5*^*−/−*^ mice exhibit partially impaired peroxisomal functions, likewise, do not develop measurable thinning of the retinal layers, but were reported to show increases in INL, IPL and RGCL thickness at early ages [62, 63]. Decreased cone and bipolar cells numbers and reduced ERG scotopic a-wave as well as scotopic and photopic b-wave amplitudes, and a reactive astrogliosis were also reported, potentially suggesting a partially similar pathogenesis in this mouse model. However, unlike in *Acbd5*^*−/−*^ mice, in Pex1-G844D mice losses in cone cell significantly precede the bipolar cell degeneration, implying also differences in the pathology development. Moreover, the Pex1-G844D mice develop a severe RPE pathology with a 60% reduction in cell numbers in the ventral retina region at the age of 6 months [64]. While there is only limited data on lipid alterations in the neural retina of Pex1-G843D mice, an elaborate MSI-analysis of RPE flat mount preparations revealed significant reductions in phospholipid DHA and plasmalogens levels implying a more widespread dysfunction in peroxisomal pathways as expectable for a peroxisome biogenesis disorder [64]. In this regard, the obvious PIS/POS pathology in Pex1-G843D mice might primarily result from a potential general shortage of DHA in the outer retinal layers. Since *Acdb5*^*−/−*^ mice did not exhibit significant alteration in DHA or plasmalogens, the milder retinal phenotype might be most likely attributed to the accumulation of VLCFA-PUFA, which is also characteristic for the retina-specific *Cre-Mfp2*^*−/−*^ mouse. Interestingly, the MALDI-MSI analysis of the Pex1-G843D mice revealed early differential regional membrane lipid alterations in the RPE, which correlated with the more severe RPE pathology in the ventral part of the retina [64]. At the late stage of one year, we did observe regional layer-specific differences in the accumulation of extremely long VLC-PUFA (> C32) but not across the transect of the retina from *Acbd5*^*−/−*^ mice. However, we cannot exclude, that those or other lipid species might show such two-dimensional spatial changes at earlier time points, which should be subject of future studies on the retinal pathogenesis in ACBD5-deficient mice.

As mentioned above, a deficiency in ACBD5 leads only to reduced peroxisomal β-oxidation activities [18, 19]. In the light of the studies characterizing a complete peroxisomal β-oxidation deficiency, the retinal pathology observed in the *Acbd5*^*−/−*^ model maybe interpreted as follows:

1. The retinae of *Acbd5*^*−/−*^ mice do not exhibit reductions in phospholipid DHA levels, which would explain the late onset of the retinal pathogenesis and the relatively mild phenotype observed for the photoreceptor cells. Since retinal DHA is in addition to nutrition mainly supplied by the liver [65], the massive proliferation of peroxisomes in hepatocytes of *Acbd5*^*−/−*^ mice appears to a significant extent compensate the reduced peroxisomal β-oxidation activities [23]. In this respect, plasma DHA-concentrations of 70% in *Acbd5*^*−/−*^ mice should be sufficient to supply especially the outer retina via the blood.
2. As shown by MALDI MS imaging, the remaining peroxisomal β-oxidation in the RPE of *Acbd5*^*−/−*^ mice seems to be still able to allow sufficient VLC-PUFA degradation to prevent a massive disruption of membrane lipid homeostasis in the RPE. Nevertheless, the increased number of enlarged and multinucleated cells points to degenerative changes in the RPE mirrored by the disruption of the membrane basal fold architecture. The latter might indicate progressively dysfunctional metabolite exchange between the RPE and the underlying choroidea, which eventually might affect POS homeostasis. However, future studies are required to decipher specifically the RPE’s contribution to the retinal pathology in *Acbd5*^*−/−*^ mice.
3. VLC-PUFA are locally increased in PC of the inner layers of *Acbd5*^*−/−*^ retinae. ACBD5, as an acyl-CoA binding protein, is not only postulated to support VLCFA import into peroxisomes but also facilitates membrane contacts between peroxisomes and the ER – potential hubs for the delivery of DHA-synthesis pathway intermediates to peroxisomes. The outer retinal layers appear to receive DHA preferentially from external sources (nutrition, liver) via the blood. By contrast, the inner retinal layers, which according to our MALDI-MSI analysis exhibit also substantial amounts of DHA-containing PC, might rely on its intrinsic synthesis to guarantee normal membrane lipid DHA levels. A dysfunctional delivery of DHA precursors from the ER to peroxisomes could lead to reduced DHA synthesis rates and as a result to a compensatory induction of the ER FA elongation and desaturation system [66, 67] in order to provide more n3-C(24:6) for peroxisomal β-oxidation. In ACBD5-deficient tissue, the thereby increased supply with n3-C(24:6) could compensate for the reduced peroxisomal VLCFA import/chain-shortening capacities resulting in normal DHA concentration in membrane lipids. Simultaneously, however, cytosolic concentrations of pathway intermediates, which serve as substrates for ER ELOVLs, would rise. This results in increased FA elongation and subsequent accumulation of VLC-PUFA in PC as observed in the plexiform layers of the *Acbd5*^*−/−*^ retina. Both pre- and postsynaptic membranes require a specialized lipid composition in order to maintain their highly specific membrane protein architecture [68, 69]. Increases in VLC-PUFA in bipolar cells could alter membrane fluidity at postsynaptic side of photoreceptor ribbon synapse terminals and/or presynaptic membranes and synaptic vesicles. Both could disrupt physiological synaptic signal transmission and eventually lead to retinal cell degeneration. Our observation of morphologically altered photoreceptor ribbon synapses in *Acbd5*^*−/−*^ mice confirm changes in the correspondent synaptic architecture and suggest that altered bipolar cell physiology may contribute to the development of the retinal pathology.
4. The retina of *Acbd5*^*−/−*^ mice exhibits a gliosis of astrocytes and Müller cells and microgliosis represented by the migration of microglia into the outer retinal layers and subretinal space. Müller cells, spanning the whole neural retina, are key players in maintaining retinal homeostasis [70]. Hence, their activation by physiologically malfunctioning neural cells (e.g. bipolar cells) might spread the pathology from the inner retinal layers to regions, which do not exhibit pathological accumulations of VLC-PUFAs. Microglia uniquely express extracellular lipid sensors [71], which might be sensitive to the accumulation of VLC-PUFAs in membranes from neural cells of the inner retina. Once activated, microglia migration towards the outer retinal layers might likewise support pathological reaction in these regions. Moreover, PUFAs are educts for numerous inflammatory mediators. Commonly, arachidonic acid is substrate for the synthesis of proinflammatory prostaglandins, thromboxanes and leukotrienes, whereas EPA and DHA are required for the synthesis of the pro-resolving, anti-inflammatory lipid mediators resolvins, protectins and maresins [72]. An increase in VLC-PUFA, caused by dysfunctional peroxisomal β-oxidation may lead to a FA spectrum shifting the equilibrium from anti- to pro-inflammatory mediators thereby promoting activation of microglia and/or astroglia. As a result, inflammatory responses might promote secondary pathogenesis in retinal layers, which are not a primary target of the lipid metabolic alterations initially induced by the loss in ACBD5 function.

In this work, we show that the loss of ACBD5 induces a locally restricted accumulation of extremely long VLC-PUFA (> C32) in PC of the mouse retina. Moreover, we observed that differences in the cellular phospholipid FA composition of the retina are reflected by the degree of saturation and chain length of the accumulating FA species, which can be explained by the cell-specific expression of distinct sets of substrate-specific FA elongases and desaturases [73]. Most importantly, VLCFA neither accumulate homogenously in the whole retina nor specifically in regions with the highest described natural VLC-PUFA content. According to our data the complex disturbance in lipid homeostasis in the retina of *Acbd5*^*−/−*^ mice is evoked by the interplay between regional, tissue-intrinsic, and extrinsic capacities of FA acid metabolism and corresponding compensatory responses and is therefore a suitable model to unravel general principles in the pathogenesis of lipid metabolic disorders. According to the very limited number of ERG analyses, the retinal phenotype from RDLKD patients was alternatively described as either a cone rod or rod cone dystrophy [17]. Hence, as in the *Acbd5*^*−/−*^ model both light receptive pathways are dysfunctional in humans, but presumably with a considerable variation in phenotype. So far, the only patient OCT published, revealed a severe maculopathy implying a more severe degeneration of cone photoreceptor cells [15], which correlates with the significant decline in cones found in *Acbd5*^*−/−*^ mice in this study. However, the focus on the photoreceptor phenotype in reports from RDLKD patients – whilst data on ERG b-waves have not yet been published – might suggest that the human disorder is characterized by a more prominent photoreceptor dysfunction. Nevertheless, ACBD5-deficient mouse models show principally a similar but somewhat less severe pathology, potentially caused by an efficient compensatory peroxisome proliferation in liver supplying the outer retina with DHA. In this respect ACBD5-deficient mice offer the opportunity to decipher fundamental molecular alterations responsible for the retinal dysfunction. This in turn could help to unravel the pathogenesis of RDLKD and to develop therapies for afflicted humans.

## Supplementary Information

Below is the link to the electronic supplementary material.

Supplementary Material 1

Supplementary Material 2

## Data Availability

All data generated and analysed during the current study are available from the corresponding author upon request.
