# Supplementary material for: Local accumulation of very long-chain PUFA in plexiform layers associates with retinal dysfunction in a mouse model of peroxisomal ACBD5-deficiency
Source: Cell Mol Life Sci. 2025 Dec 1;83(1):26. doi: 10.1007/s00018-025-05971-8 (PMC12775220; doi:10.1007/s00018-025-05971-8)
Supplement: Supplementary file 2 — Supplementary Material 2 [file 18_2025_5971_MOESM2_ESM.pdf]

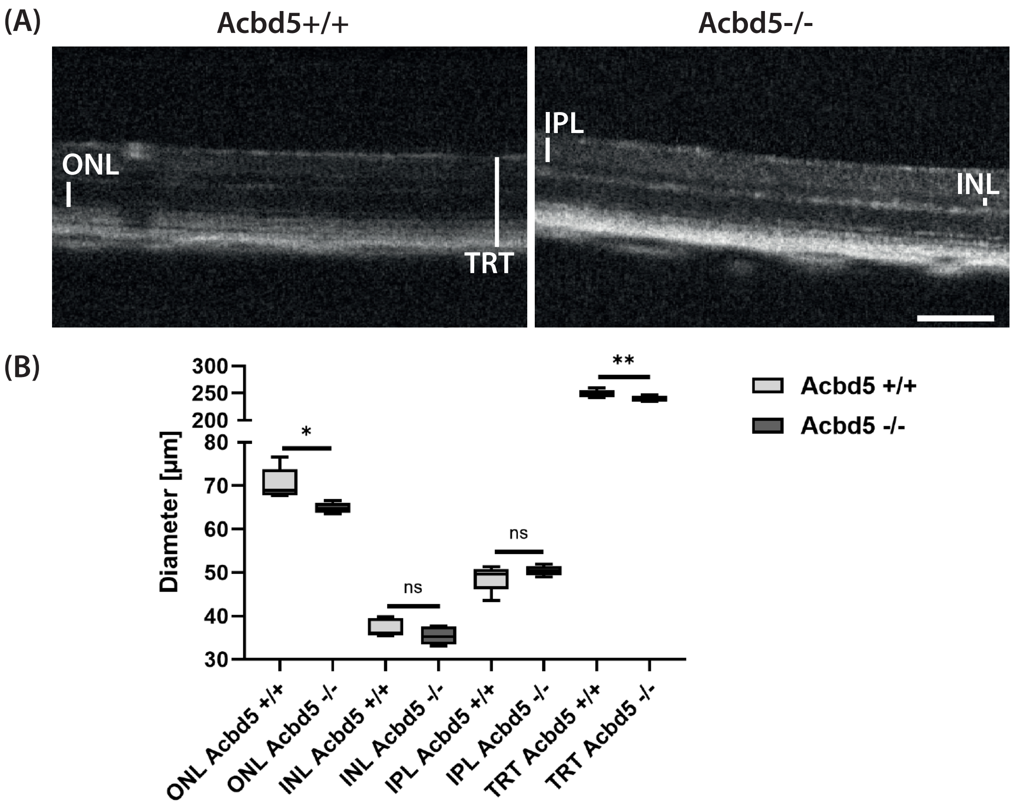

Fig. S1: (A) Representative optical coherence tomograms (OCT) used to determine retinal thickness *in vivo*. (B) Quantification of the total retinal thickness (TRT) and diameters for outer (ONL), inner nuclear layer (INL) and inner plexiform layer (IPL) in OCTs from *Acbd5*$^{-/-}$ and *Acbd5*$^{+/+}$ mice. ns – not significant, * $p < 0.05$, ** $p < 0.01$

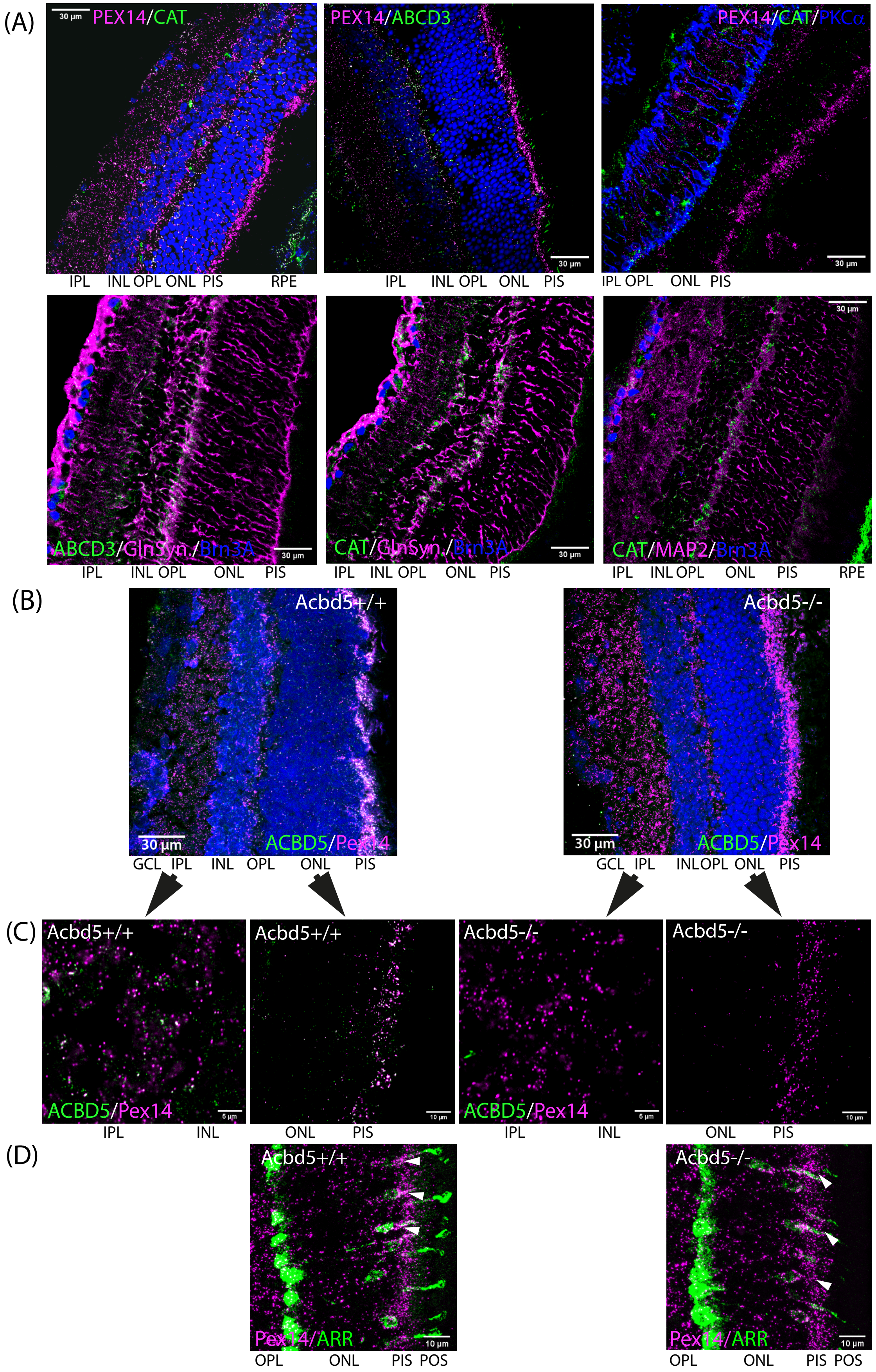

Fig. S2: Peroxisomes in *Acbd5*$^{-/-}$ and *Acdb5*$^{+/+}$ mice. To allow identifcation of the antigen IF signals, respective protein names are depicted in the same colour in each image (TOPRO as a marker for nuclei is shown in blue). (A) IF images of different peroxisomal proteins in adult *Acdb5*$^{+/+}$ mice; catalase (CAT) and ABCD3 are examples for proteins involved in peroxisomal metabolic tasks, while Pex 14 as a peroxin illustrates the general distribution of peroxisomes in the retina. (B) Overview of ACBD5 IF signal distribution in the retina of *Acbd5*$^{-/-}$ and *Acdb5*$^{+/+}$ mice. (C) Magnifications of selected areas from B. (D) Peroxisome distribution in photoreceptor cells; note that cone PIS labelled by cone arrestin (ARR) exhibit higher peroxisome densities than rod PIS. Abbr.: PIS - photoreceptor inner segments, ONL - outer nuclear layer, OPL - outer plexiform layer, INL - inner nuclear layer, IPL - inner plexiform layer, GCL - ganglion cell layer, RPE - retinal pigment epithelium

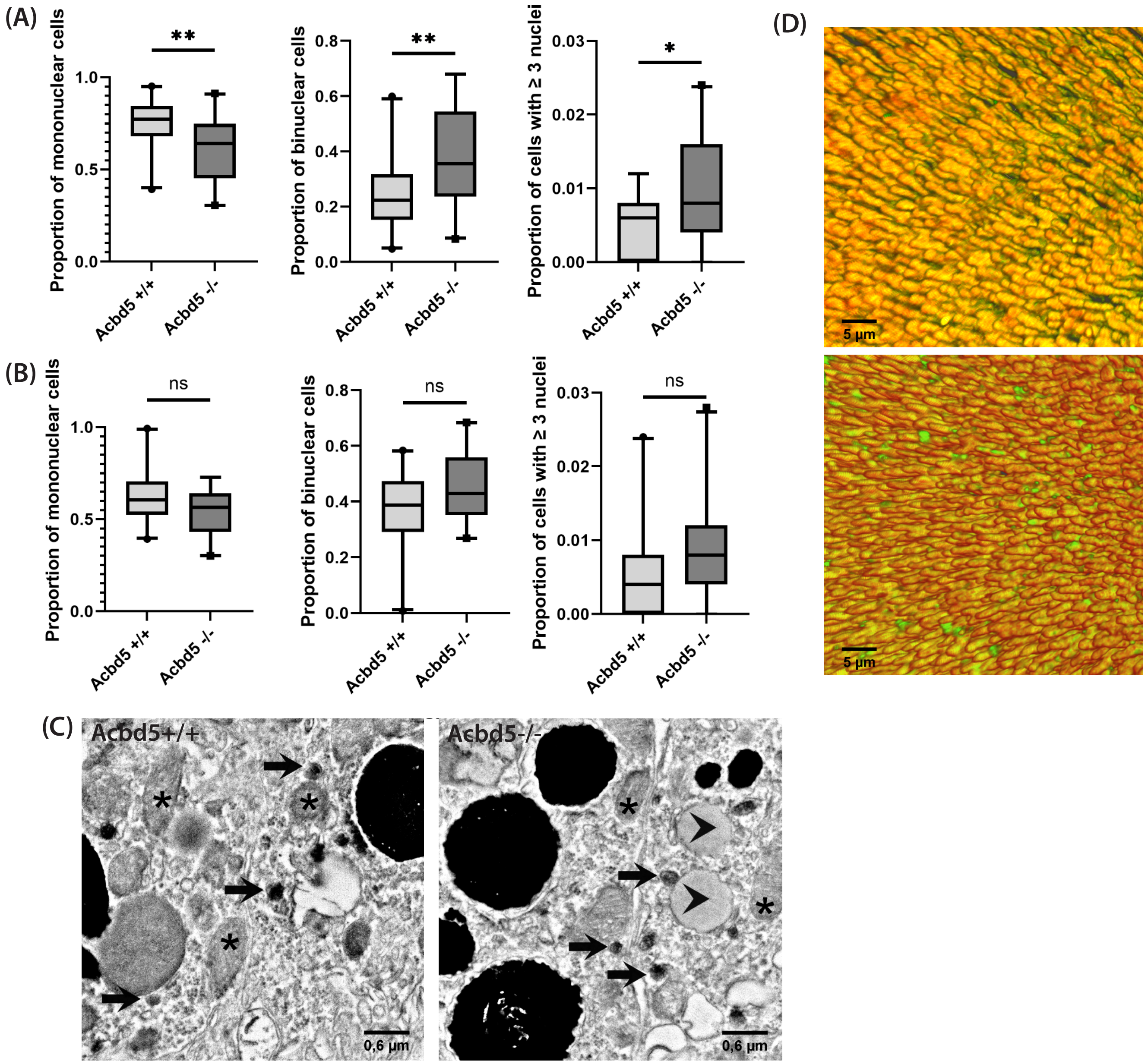

Fig. S3: (A) Quantification of mono-, bi- and multinucleated RPE cells in 3-month-old animals (n=5 per genotype). (B) Quantification of mono-, bi- and multinucleated RPE cells in 12-month-old animals (n=5 per genotype). (C) Peroxisomes identified by alkaline DAB staining in RPE cells, mitochondria ere indicated by stars, lipid droplets (LD) by arrowheads, and peroxisomes by arrows. Note the vicinity of peroxisomes to LD in the *Acbd5*$^{-/-}$ specimen. (E) 3D-reconstrution of the apical microvilli from RPE flat mounts stained with Nile-Red.

Lysophosphatidylcholine species

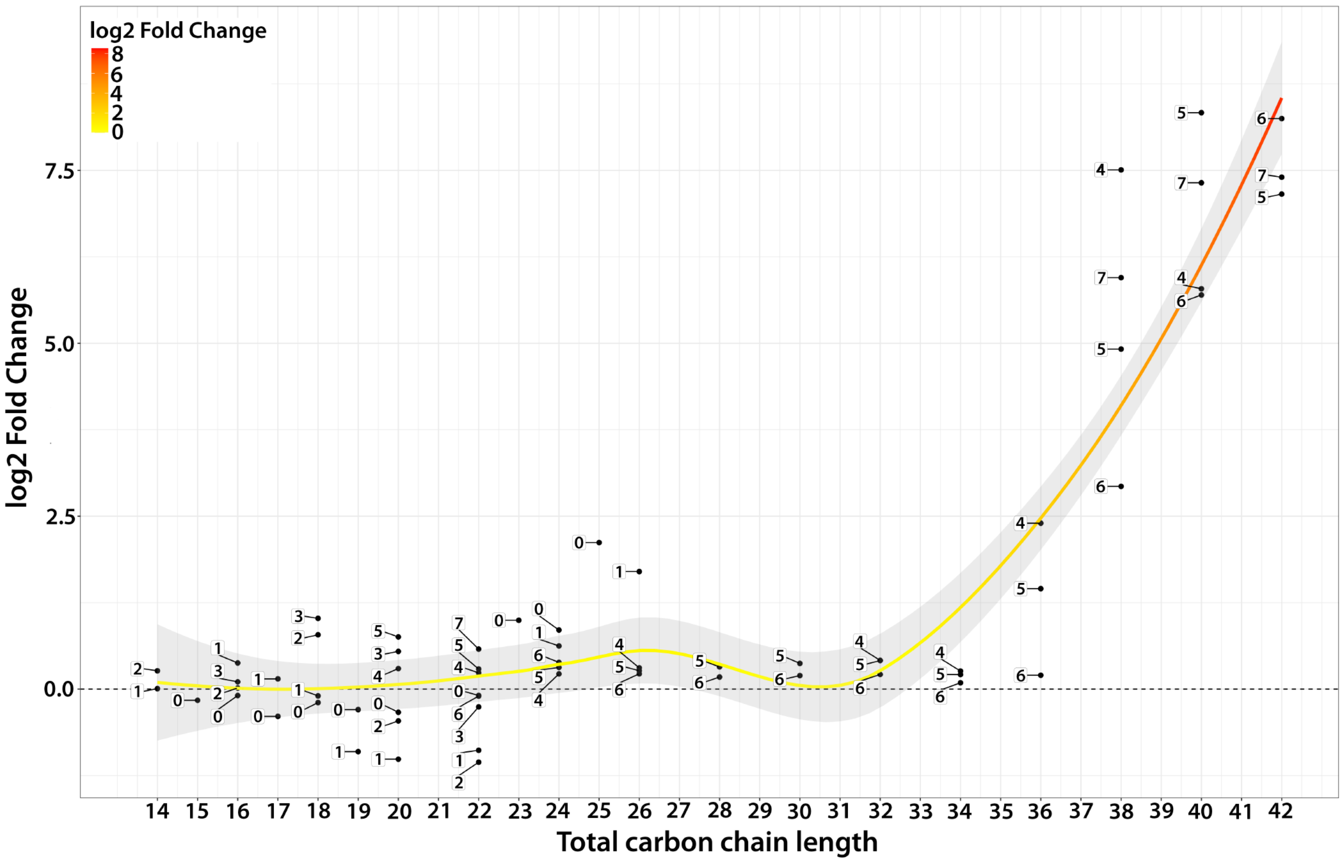

Cholesterol ester species

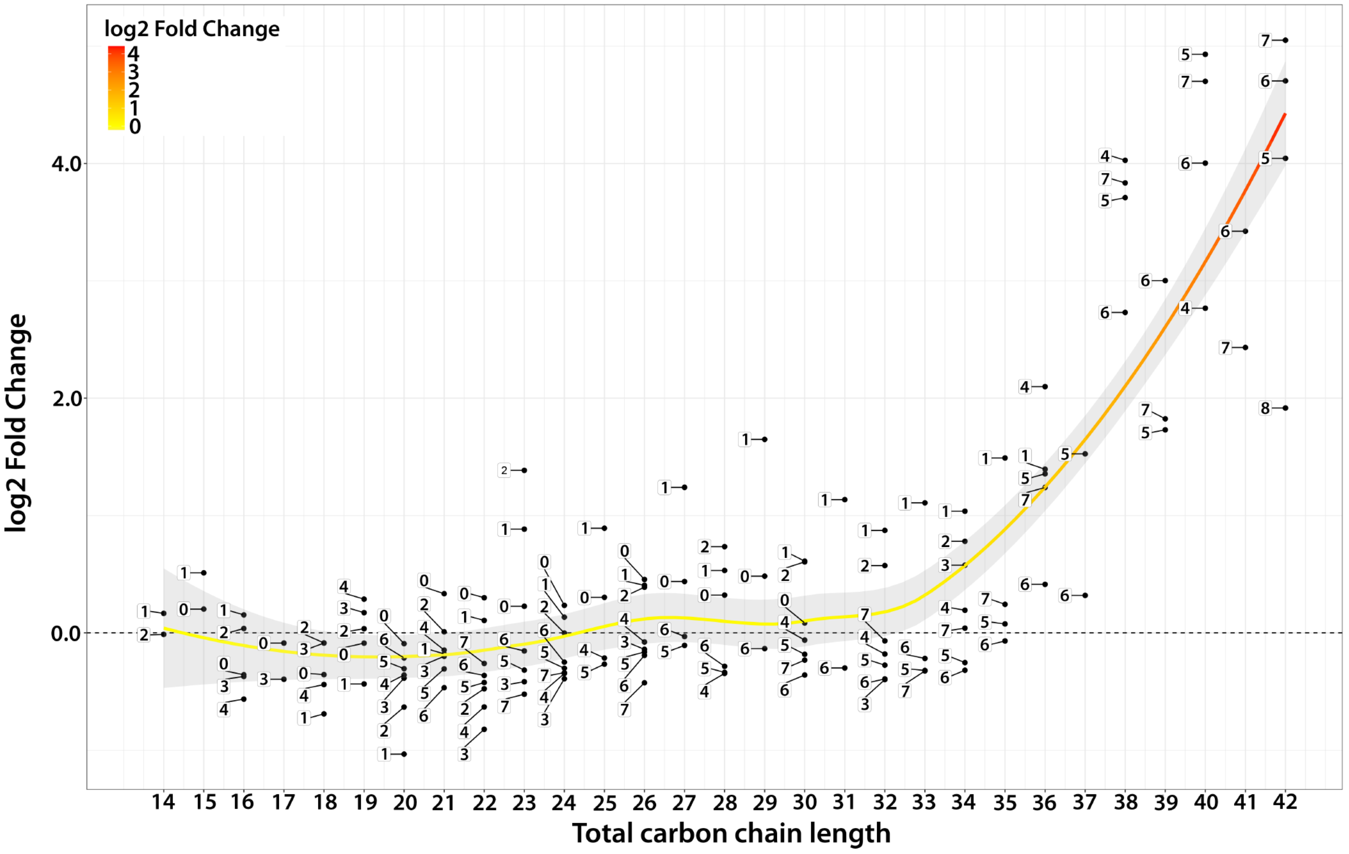

Fig. S4: Changes in distinct lysophosphatidylcholine (LPC) and cholesterol ester (CE) species depicted on a log2 scale against. FA chain length. The numbers in the rectangles show the number of double bonds of individual lipids with the same FA chain length.

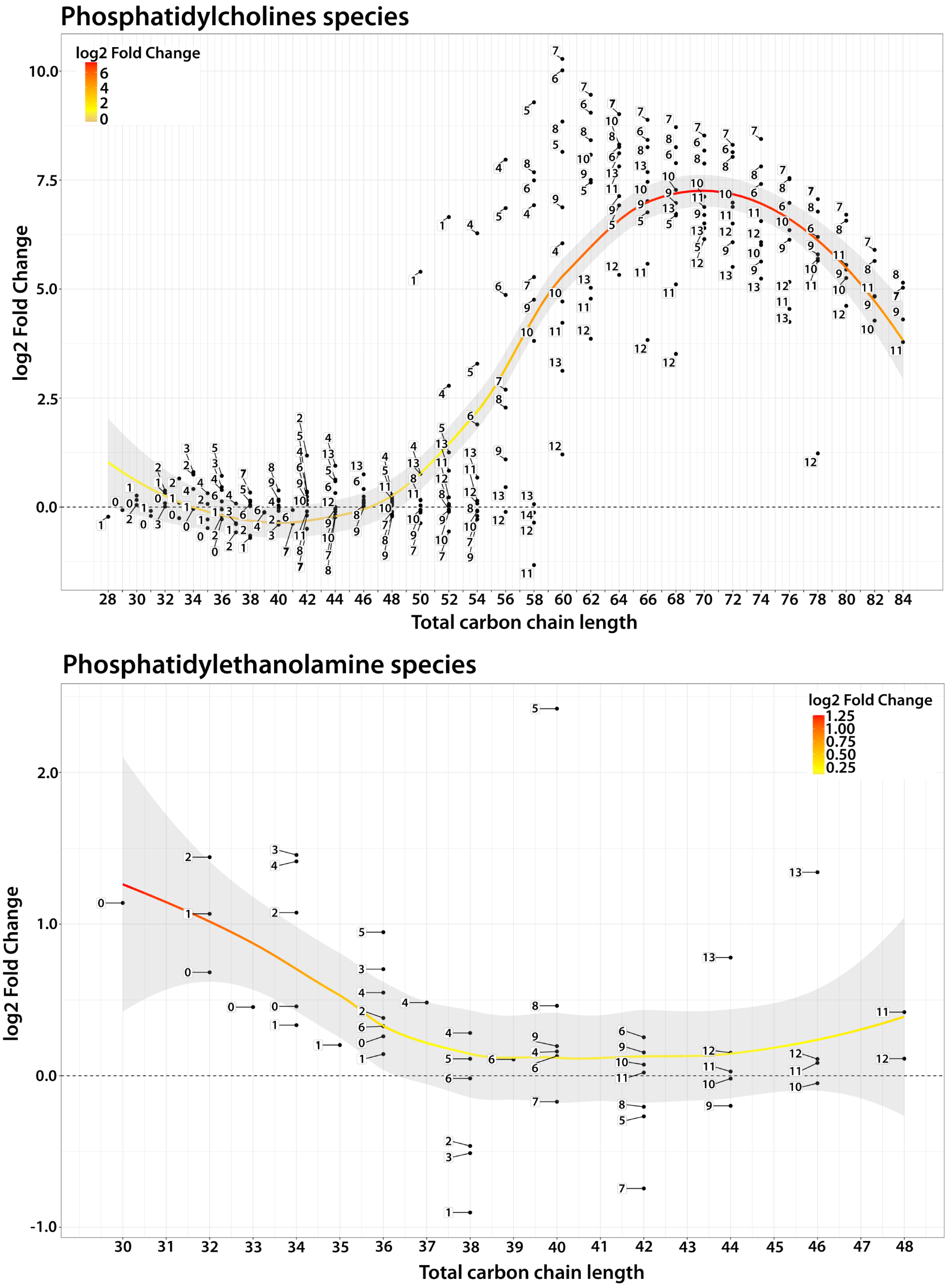

Fig. S5: Changes in distinct phosphatidylcholine (PC) and phosphatidylethanolamine (PE) species depicted on a log2 scale against FA chain length. The numbers in the rectangles show the number of double bonds of lipids with the same total chain length.

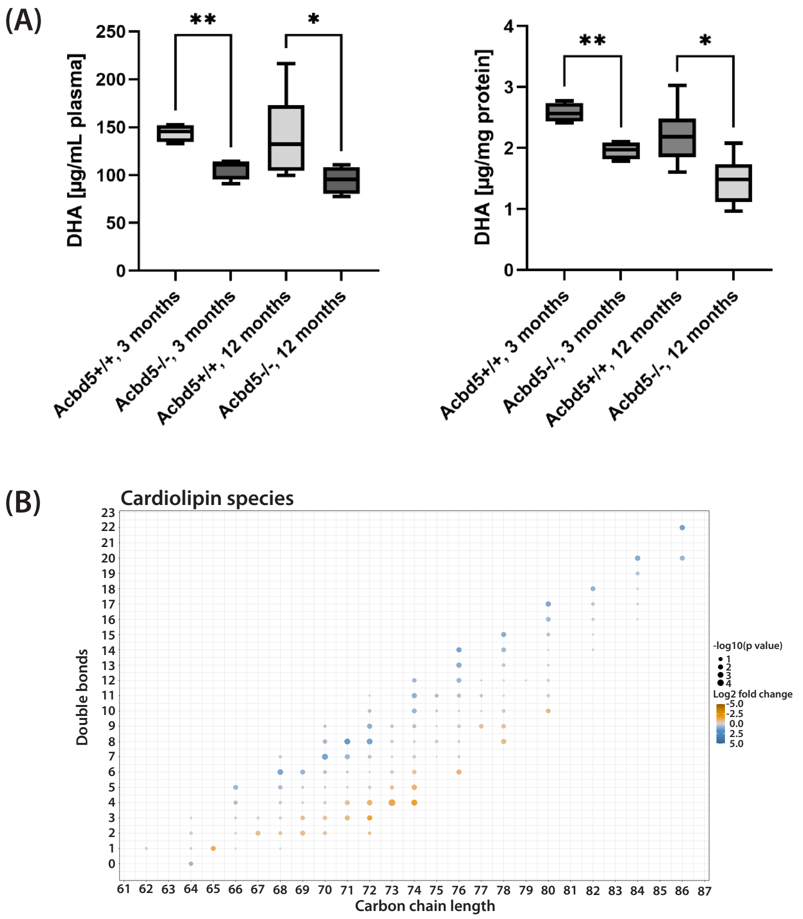

Fig. S6: Supplemental information on lipid alterations in *Acbd5*$^{-/-}$ mice: (A) DHA concentrations in blood plasma from 3- and 12-month-old *Acbd5*$^{-/-}$ mice (3 months: n=4/genotype, 12 months: n=6/genotype). **(B)** Fatty acid (FA) composition of cardiolipins compared between *Acbd5*$^{-/-}$ and *Acbd5*$^{+/+}$ retina homogenates; circles in blue show lipid species elevated and circles in orange lipid species decreased in *Acbd5*$^{-/-}$ retinae, circle size signifies the measure of significance of the alteration, the x-axis depicts the FA carbon chain length, the y-axis the number of double bonds/FA.

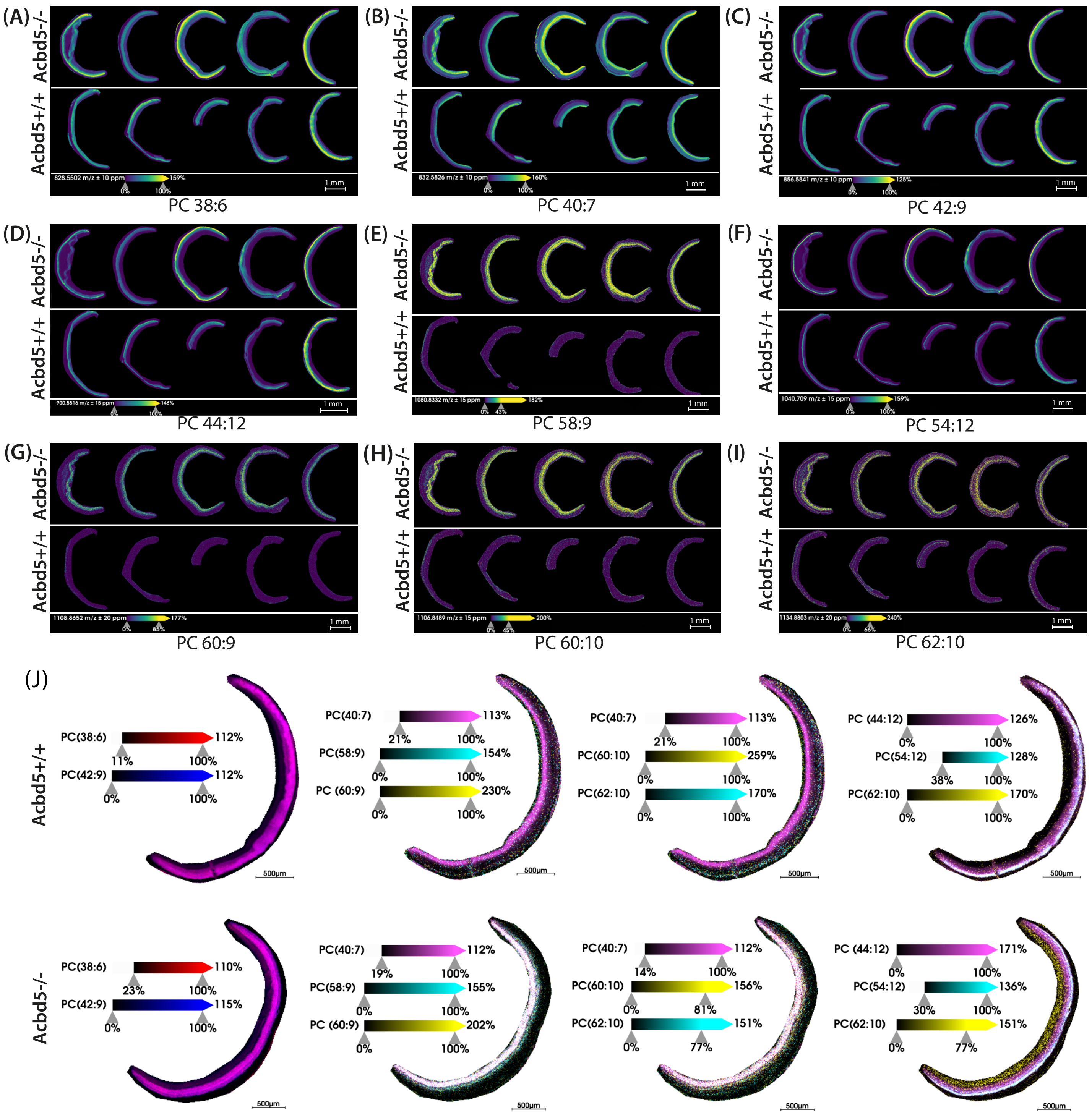

Fig. S7: Supplementary information on local lipid alterations observed in *Acbd5*$^{-/-}$ mice by MALDI-MS Imaging. (A-I) Distribution of distinct phosphatidylcholine (PC) species in *Acbd5*$^{-/-}$ and *Acbd5*$^{+/+}$ mice, each cross-section shown was derived from an individual specimen (n = 5/genotype); distinct PC species were identified by MALDI-MS imaging according to their m/z ratios; their intensity distribution is visualized by a range of false colors depicted in the scale below the images. Note that PC(58:9) (m/z 1080.83, [M+H]+, mass error 2.04 ppm), PC(60:9) (m/z 1108.87, [M+H]+, mass error 1.44 ppm), PC(60:10) (m/z 1106.85, [M+H]+, mass error 1.99 ppm) and PC(62:10) (m/z 1134.88, [M+H]+, mass error 1.85 ppm) is specifically and consistently accumulated in the inner layers of the *Acbd5*$^{-/-}$ retina. PC(38:6) (m/z 828.55, [M+Na]+, mass error 1.45 ppm), PC(40:7) (m/z 832.58, [M+H]+, mass error 3.0 ppm), PC(42:9) (m/z 856.58, [M+H]+, mass error 1.17 ppm) and PC(54:12) (m/z 1040.71, [M+Na]+, mass error 1.06 ppm), PC(44:12) (m/z 900.55, [M+Na+], mass error 1.15 ppm are equally distributed in *Acbd5*$^{-/-}$ and *Acbd5*$^{+/+}$ mice. (J) Overlays of respective lipid spectra allow the localisation of lipid species by comparison to PC(38:6), PC(40:7), PC(54:12) and PC(60:10), which were localised by alignement to correspondent, consecutive HE-stainings shown in Fig. 7.

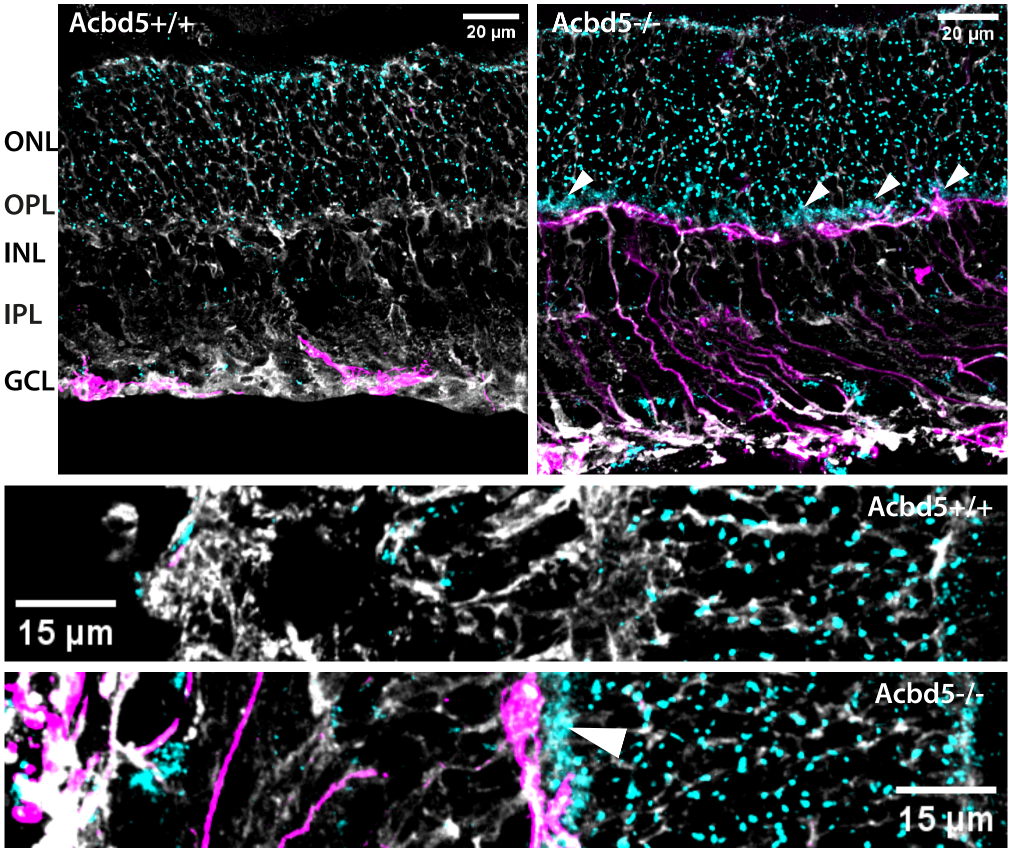

Fig. S8: Lysosome distribution in retinal cross sections from *Acbd5*$^{-/-}$ and *Acbd5*$^{+/+}$ mice. Lysosomes were marked with antibodies against LAMP1 (cyan), Müller cells are identified by antibodies directed against glutamine synthetase (grey) and GFAP (magenta). Note that big cyan spots colocalize to radial Müller cell projections, while in *Acbd5*$^{-/-}$ retinae smaller LAMP1-positive puncta (arrowheads) accumulate in the central region of the outer plexiform layer (OPL). Abbr.: ONL - outer nuclear layer, INL -. inner nuclear layer, IPL - inner plexiform layer, GCL - ganglion cell layer

Table S1: Primary antibodies

Antigen	Species	Dilution	Source
ABCD3	ms	1:500	Sigma-Aldrich, St. Louis, Missouri, USA
ACBD5	rb	1:100	Proteintech Germany GmbH, Planegg-Martinried, DE
BRN3A	gt	1:200	Santa Cruz Biotechnolgy Inc., Dallas, Texas, USA
Calbindin	gp	1:1000	Synaptic Systems GmbH, Göttingen, DE
Catalase	ms	1:200	Abcam Limited, Cambridge, UK
Cone Arrestin	rb	1:1000	Sigma-Aldrich, St. Louis, Missouri, USA
Cone Opsin	rb	1:1000	Sigma-Aldrich, St. Louis, Missouri, USA
GFAP	ms	1:1000	Santa Cruz Biotechnolgy Inc., Dallas, Texas, USA
Glutamine synthetase	rb	1:1000	Thermo Fisher Scientific Inc., Waltham, Massachusetts, USA
IBA1	rb	1:1000	Fujifilm Wako Chemicals Europe GmbH, Neuss, DE
LAMP1	rt	1:200 (RPE)<br>1:500 (Retina)	Santa Cruz Biotechnolgy Inc., Dallas, Texas, USA
MAP2	rb	1:100	Synaptic Systems GmbH, Göttingen, DE
NeuN	gp	1:1000	Sigma-Aldrich, St. Louis, Missouri, USA
PEX14	gp	1:2000	Proteogenix (7790-03107-A02, costum made)
PEX14	rb	1:2000	Gift from D. Crane, University of Brisbane, Australia
PKCα	gt	1:1000	Santa Cruz Biotechnolgy Inc., Dallas, Texas, USA
Rhodopsin	rb	1:1000 (Retina)<br>1:2000 (RPE)	Antibodies-online GmbH, Aachen, DE
CTBP2 (Ribeye)	ms	1:500	Synaptic Systems GmbH, Göttingen, DE

ch: chicken; GFAP: Glial Fibrillary Acidic Protein; gp: guinea pig; gt: goat; BRN3A: Brain-specific homeobox 3A IBA1: Ionized Calcium-binding Adapter Molecule 1; LAMP1/ 2: Lysosomal-associated Membrane Protein-1/ 2; MBP: Myelin Basic Protein; MAP2: Microtubule-associated protein 2, ms: mouse; NeuN: neuronal nucleus antigen; PEX 14: Peroxin 14; PKCα: Proteinkinase C α; rb: rabbit; rt: rat

Table S2: Secondary antibodies

Target-species	Target Ig-class	host-species	Fluorescence conjugate	Dilution	Source
ch	IgG	gt	Alexa Fluor® 637	1:500	LifeTechnologies – Thermo Fisher Scientific Inc., Waltham, Massachusetts, USA
gp	IgG	gt	Alexa Fluor® 568	1:1000	LifeTechnologies – Thermo Fisher Scientific Inc., Waltham, Massachusetts, USA
gp	IgG	dk	Alexa Fluor® 637	1:500	LifeTechnologies – Thermo Fisher Scientific Inc., Waltham, Massachusetts, USA
gt	IgG	dk	Alexa Fluor® 568	1:1000	LifeTechnologies – Thermo Fisher Scientific Inc., Waltham, Massachusetts, USA
ms	IgG	rb	Alexa Fluor® 568	1:1000	LifeTechnologies – Thermo Fisher Scientific Inc., Waltham, Massachusetts, USA
rb	IgG	dk	Alexa Fluor® 488	1:1000	LifeTechnologies – Thermo Fisher Scientific Inc., Waltham, Massachusetts, USA
rb	IgG	gt	Alexa Fluor® 568	1:1000	LifeTechnologies – Thermo Fisher Scientific Inc., Waltham, Massachusetts, USA
rb	IgG	gt	Alexa Fluor® 637	1:500	LifeTechnologies – Thermo Fisher Scientific Inc., Waltham, Massachusetts, USA
rt	IgG	dk	Alexa Fluor® 488	1:1000	LifeTechnologies – Thermo Fisher Scientific Inc., Waltham, Massachusetts, USA

ch: chicken; dk: donkey; gp: guinea pig; gt: goat; ms: mouse; rb: rabbit; rt: rat

Table S3: Statistical parameters for selected phosphatidylcholines analysed by MALDI-MSI

m/z Feature	Lipid Species	P-value	P-value adjusted	Fold change
828.55	PC 38:6	0.3779359462	0.5039145949	1.2
832.58	PC 40:7	0.1207213023	0.1931540837	1.3
856.58	PC 42:9	0.7216111835	0.7216111835	1.1
900.55	PC 44:12	0.9773741995	0.9773741995	1.0
1040.71	PC 54:12	0.4670333449	0.5337523942	1.2
1080.83	PC 58:9	0.0001632359	0.0003264718	6.5
1106.85	PC 60:10	0.0001033931	0.0002943229	4.4
1108.87	PC 62:9	0.0001103711	0.0002943229	6.2
1134.88	PC 62:10	0.0001051590	0.0002943229	3.4
